# Supplementary material for: Versatile human cardiac tissues engineered with perfusable heart extracellular microenvironment for biomedical applications
Source: Nat Commun. 2024 Mar 22;15:2564. doi: 10.1038/s41467-024-46928-y (PMC10960018; doi:10.1038/s41467-024-46928-y)
Supplement: Supplementary file 1 — Supplementary Information [file 41467_2024_46928_MOESM1_ESM.pdf]

Supplementary Information

Versatile human cardiac tissues engineered with perfusable heart extracellular microenvironment for biomedical applications

Sungjin Min[1#], Suran Kim[1,2#], Woo-Sup Sim[3,4#], Yi Sun Choi[1], Hyebin Joo[1], Jae-Hyun Park[3,4], Su-Jin Lee[5], Hyeok Kim[3,4], Mi Jeong Lee[1], Inhea Jeong[6], Baofang Cui[1], Sung-Hyun Jo[7], Jin-Ju Kim[3,4], Seok Beom Hong[8], Yeon-Jik Choi[9], Kiwon Ban[10], Yun-Gon Kim[7], Jang-Ung Park[6,11,12,13], Hyang-Ae Lee[5], Hun-Jun Park[3,4,14]*, Seung-Woo Cho[1,2,12,13]*

[1]Department of Biotechnology, Yonsei University, Seoul 03722, Republic of Korea
[2]Cellartgen, Seoul 03722, Republic of Korea
[3]Department of Biomedicine & Health Sciences, College of Medicine, The Catholic University of Korea, Seoul 06591, Republic of Korea
[4]Division of Cardiology, Department of Internal Medicine, Seoul St. Mary's Hospital, The Catholic University of Korea, Seoul 06591, Republic of Korea
[5]Department of Predictive Toxicology, Korea Institute of Toxicology, Daejeon 34114, Republic of Korea
[6]Department of Materials Science and Engineering, Yonsei University, Seoul 03722, Republic of Korea
[7]Department of Chemical Engineering, Soongsil University, Seoul 06978, Republic of Korea
[8]Department of Thoracic and Cardiovascular Surgery, Seoul St. Mary's Hospital, College of Medicine, The Catholic University of Korea, Seoul 06591, Republic of Korea
[9]Division of Cardiology, Department of Internal Medicine, Eunpyeong St. Mary's Hospital, College of Medicine, The Catholic University of Korea, Seoul 03312, Republic of Korea
[10]Department of Biomedical Sciences, City University of Hong Kong, Kowloon 999077, Hong Kong
[11]Department of Neurosurgery, Yonsei University College of Medicine, Seoul 03722, Republic of Korea
[12]Center for Nanomedicine, Institute for Basic Science (IBS), Seoul 03722, Republic of Korea
[13]Graduate Program of Nano Biomedical Engineering (NanoBME), Advanced Science Institute, Yonsei University, Seoul 03722, Republic of Korea
[14]Cell Death Disease Research Center, College of Medicine, The Catholic University of Korea, Seoul 06591, Republic of Korea

[#]S. Min, S. Kim, and W. S. Sim equally contributed to this work.

*To whom correspondence should be addressed: seungwoocho@yonsei.ac.kr or cardioman@catholic.ac.kr

Supplementary Methods

Characterization of heart extracellular matrix (HEM)

The amounts of dsDNA, glycosaminoglycan (GAG), and collagen in decellularized heart tissues were quantified and compared with those of native heart tissues. dsDNA from lyophilized HEM and native heart tissues was extracted using a DNA extraction kit (#9765A, TaKaRa, Shiga, Japan) according to the manufacturer's instructions. The GAG content in HEM and native heart tissues was determined using a 1,9-dimethylmethylene blue (DMMB) assay (Sigma-Aldrich, St. Louis, MO, USA) with a modification from a previous protocol[1]. Lyophilized HEM and native heart tissues were digested with 150 μg/ml papain (Sigma-Aldrich) and 10 mM N-acetyl-L-cysteine (Sigma-Aldrich) at 60°C overnight. Chondroitin sulfate (Sigma-Aldrich) was used as a standard with serial dilutions from 40 μg/ml. After adding DMMB solution, the GAG content in HEM and native tissues was quantified by measuring absorbance at 525 nm on a microplate reader (Tecan, Männedorf, Switzerland). The amount of collagen in HEM and native tissues was assessed using a Sircol™ Soluble Collagen Assay (Biocolor, Carrickfergus, UK) following the manufacturer's protocols.

Characterization of HEM hydrogels

The viscoelastic properties of the hydrogels were examined using a rotating rheometer (MCR 102, Anton Paar, Graz, Austria) with an 8 mm parallel plate. A frequency sweep mode was performed to measure the storage moduli (G') and loss moduli (G'') of the hydrogels across a frequency of 0.1 to 10 Hz at a constant strain of 1%. The elastic moduli of the hydrogels were obtained based on the storage moduli of hydrogels measured at a frequency of 1 Hz.

To examine the internal architecture of the HEM hydrogels, the samples were fixed with 10% neutral buffer formalin (Thermo Fisher Scientific, Waltham, MA, USA) for 10 min and subjected to dehydration with a progressively increasing concentration of ethanol as

follows: 50%, 60%, 70%, 80%, 90%, and 100%. Subsequently, the hydrogels were immersed in tert-butyl alcohol (TCI, Tokyo, Japan) and stored at –80°C. For the imaging with field emission scanning electron microscopy (FE-SEM), the frozen HEM hydrogels were lyophilized using a freeze dryer (FDU-2100, Eyela, Tokyo, Japan) under a vacuum pressure of 8.5 Pa and a trap temperature of –85°C. The internal architecture of the hydrogels was observed using FE-SEM (JSM-7800F, JEOL Ltd., Tokyo, Japan).

In vitro and _in vivo_ biocompatibility evaluation of HEM

In vitro immunogenicity was assessed by examining the levels of inflammatory cytokines released from macrophages co-cultured with HEM hydrogels. Murine RAW 264.7 macrophage cells were seeded in 24-well plates at a density of 1.0×10^6 cells/well and cultured in Dulbecco's Modified Eagle Medium (DMEM) containing 10% fetal bovine serum (FBS, Thermo Fisher Scientific) and 1% penicillin-streptomycin (PS) for 24 h at 37°C. Medium in each well was replaced with fresh medium the following day. For co-culture with HEM hydrogel, 50 µl of 5 mg/ml HEM hydrogel was placed on Transwell inserts (Corning, Corning, NY, USA) in each well. Fresh medium with or without 1 µg/ml lipopolysaccharide (LPS, Sigma-Aldrich) was utilized as positive and negative controls, respectively. Supernatants were collected after 6 h of incubation. The secretion levels of tumor necrosis factor-alpha (TNF-α) were determined by enzyme-linked immunosorbent assay (ELISA) using a mouse TNF-α ELISA kit (R&D Systems, Minneapolis, MN, USA) following the manufacturer's instructions. Endotoxin testing of HEM hydrogels was performed using a Pierce™ Chromogenic Endotoxin Quant Kit (Thermo Fisher Scientific) following the manufacturer's protocol.

The *in vivo* biocompatibility of the HEM hydrogels was evaluated by subcutaneous injection of 200 µl of 5 mg/ml HEM into BALB/c nude mice (CAnN.Cg-Foxn1 nu/CrlOri, 4-

week-old male, 20 g; Orient Bio Inc., Seongnam, Korea). Only male mice were employed to minimize variation of results according to biological sex differences.[2] These experiments were approved by the Institutional Animal Care and Use Committee (IACUC) of the Yonsei Laboratory Animal Research Center (YLARC) (permit number: IACUC-A-202111-1373-04). All mice were maintained in the housing condition with a temperature of 21 ± 2 °C, a humidity of 50 ± 10%, ventilation of 10–15/h, the light of 150–300 Lux, and noise of less than 60 dB. After 1, 4, or 7 days post-injection, mice were sacrificed and skin tissues adjacent to the hydrogels were harvested. The skin tissues were fixed with a 10% formalin solution for 12 h, washed with PBS, and embedded in paraffin. Paraffin-embedded skin samples were cut to a thickness of 6 μm and stained with hematoxylin and eosin (H&E) and toluidine blue (TB) to assess necrosis and recruitment and infiltration of immune cells at injection sites. The number of fibroblasts and immune cells at the sites of HEM injection was quantified through immunostaining for fibroblasts (α-smooth muscle actin (α-SMA) and Collagen type 1), immune cells (CD11b and CD45), M1 macrophages (inducible nitric oxide synthase (iNOS) and CD80), and M2 macrophages (CD206 and CD163). The M1/M2 ratio was calculated by dividing the number of $iNOS^+$ M1 macrophages by that of $CD206^+$ M2 macrophages or by dividing the number of $CD80^+$ M1 macrophages by that of $CD163^+$ M2 macrophages.

Protein sample preparation for mass spectrometry

Decellularized heart tissues were cut into small fragments, and proteins in the tissue samples were extracted using a Filter Aided Sample Preparation (FASP) Protein Digest kit (Abcam, Cambridge, UK) with modifications of the manufacturer's protocol. Tissue samples were reduced with 10 mM 1,4-dithiothreitol (DTT, Sigma-Aldrich) in 50 mM ammonium bicarbonate for 30 min at 60°C. Then, tissue samples were alkylated with 55 mM iodoacetamide in urea solution for 30 min in the dark and digested with trypsin (1:50 enzyme

to protein, Sigma-Aldrich) for 16 h at 37°C. The next day, digested peptide samples were centrifuged at 7,500 g for 2 min, and the resulting peptide solutions were desalted using Pierce^{TM} Peptide Desalting Spin Columns (Thermo Fisher Scientific). The desalted peptide samples were dried in a speed vacuum concentrator (Eyela) at room temperature for 5 h and resuspended in 0.1% (v/v) formic acid and 2% (v/v) acetonitrile solution. Peptide concentrations were quantified using a Pierce^{TM} Quantitative Colorimetric Peptide Assay (Thermo Fisher Scientific) and adjusted to 0.5 mg/ml.

Liquid chromatography with tandem mass spectrometry (LC-MS/MS) and data processing

Peptide samples were analyzed using an Ultimate 3000 RSLCnano System (Thermo Fisher Scientific) and Q-Exactive Orbitrap HF-X (Thermo Fisher Scientific). For peptide separation, two columns were used: Acclaim PepMap RSLC C18 (75 μm × 50 cm, filled with 2-μm C18 particles, Thermo Fisher Scientific) as the analytical column and Acclaim PepMap 100 (75 μm × 2 cm filled with 3-μm C18 particles, Thermo Fisher Scientific) as the trap column. Solvent A (0.1% formic acid) and solvent B (0.1% formic acid in acetonitrile) were applied for a 125-min LC gradient run at a constant flow of 0.270 μl/min. The LC gradient consisted of the following: equilibration to 5% solvent B (5 min), 5% to 10% solvent B (5 min), 10% to 35% solvent B (65 min), 35% to 50% solvent B (10 min), 50% to 80% solvent B for (1 min), maintenance in 80% solvent B (18 min), 80% to 5% solvent B (1 min), and equilibration at 5% solvent B (20 min). MS was operated in positive ion mode with spray voltage at 1.8 kV and capillary temperature at 250°C. A full MS scan was obtained with the following parameters: scan range of 400–2000 m/z with a full MS resolution of 60,000, automated gain control target at 3×10^6, and a maximum injection time of 100 msec. As for the MS/MS scan, parameters were set to a resolution of 15,000, automated gain control target of 1×10^5,

maximum injection time of 100 msec, normalized collision energy of 27, and dynamic exclusion of 30 sec.

Raw data from LC-MS/MS were processed using MaxQuant (2.0.3.0)[3]. MS and MS/MS spectra were searched against *Sus scrofa* (released in March 2021) in the UniProt database[4]. Label-free quantification (LFQ) was conducted with specific enzyme digestion set to trypsin/P with a maximum of two missed cleavages. Cysteine carbamidomethylation was included as a fixed modification, and methionine oxidation and N-terminal acetylation were specified as variable modifications. Peptides and proteins were filtered by setting the threshold of the peptide-to-spectrum match (PSM) false discovery rate (FDR) and protein FDR to 0.01. A match between runs was performed, and an intensity-based absolute quantification (iBAQ) algorithm was used to quantify the level of proteins present in the samples. The iBAQ represents a molar abundance of proteins within the samples.

Proteomic analysis of HEM

The protein compositions of HEM samples (A#1, A#2, A#3, B, and C) and growth factor reduced Matrigel (GFR-Matrigel) were compared by applying relative iBAQ (riBAQ). riBAQ values were calculated by dividing the iBAQ of a protein by the total protein iBAQ, referring to the relative abundance of proteins within samples. Using the Matrisome Project database[5], matrisome proteins in HEM and GFR-Matrigel were identified and categorized. Protein expression in HEM and GFR-Matrigel was compared to that of native heart tissue from the Human Protein Atlas[6]; proteins that were elevated at least 4-fold compared to other tissues were defined as heart-enriched proteins.

Functional annotation was performed using Gene Ontology Biological Processes (GOBP)[7, 8] by searching against *Sus scrofa*. The PANTHER Overrepresentation Test (Release 20210224) was used, and Fisher’s Exact statistical tests were applied with FDR correction.

Principal component analysis (PCA) and Pearson's correlation analysis were performed using GraphPad Prism 9 (GraphPad, La Jolla, CA, USA) by computing two-tailed Pearson's correlation coefficients (r) with 95% confidence intervals.

Human induced pluripotent stem cell (hiPSC) culture

The study utilizing hiPSCs was approved by the Institutional Review Board (IRB) of Yonsei University (permit number: 7001988-202004-BR-844-01E). Most experiments were performed using the KYOU-DXR0109B hiPSC line (female) obtained from the American Type Culture Collection (ATCC, Manassas, VA, USA). The GM25305 hiPSC line (LQT2 hiPSC, female) obtained from the Coriell Institute for Medical Research (Camden, NJ, USA) was used for LQTS modeling. For cardiac tissue transplantation experiments, red fluorescence protein (RFP)-expressing hiPSCs were generated using CMC-iPSC-011 (CMC11-iPSCs, male) obtained from the Catholic iPSC Research Center in the Catholic University of Korea (Seoul, Korea). All hiPSCs were cultured in cell culture plates coated with human embryonic stem cell (hESC)-qualified Matrigel (Corning) in mTeSR Plus medium (StemCell Technologies, Vancouver, Canada). hiPSCs were passaged every 5–7 days using ReLeSR (StemCell Technologies) and regularly assessed for mycoplasma contamination using a MycoAlert Mycoplasma Detection Kit (Lonza, Basel, Switzerland).

Generation of RFP-expressing hiPSCs (RFP-hiPSCs)

hiPSCs expressing RFP (tdTomato) were generated using the pEF1α-tdTomato vector (631975, Clontech, Mountain View, CA, USA). Briefly, plasmid DNA (500 ng) was transfected into hiPSCs using Lipofectamine™ Stem (STEM00001, Thermo Fisher Scientific) according to the manufacturer's instructions. Neomycin-resistant clones were isolated using selective antibiotics (10131027, Thermo Fisher Scientific). Finally, hiPSC

colonies expressing the RFP signal were sorted by flow cytometry on a FACSAria™ Fusion (BD Biosciences, San Jose, CA, USA). Purified fractions of RFP-hiPSC colonies were expanded for further differentiation into cardiomyocytes (CMs). The expression of RFP and the pluripotency of RFP-hiPSCs were analyzed on a FACSCanto II (BD Biosciences) with FlowJo software (version 10.8.1, Tree Star Inc., Ashland, OR, USA). Staining was conducted using BV421 mouse anti-Oct3/4 (565644, BD Biosciences) or BV421 mouse IgG1, k isotype control (562438, BD Biosciences).

hiPSC differentiation into cardiomyocytes (CMs) and cardiac fibroblasts (CFs)

CMs were differentiated from hiPSCs according to a reported protocol[9]. In detail, hiPSCs were dissociated and seeded in 6-well-culture plates coated with hESC-qualified Matrigel at a density of 3.5–4.5 × 10^5 cells per well in Essential 8 medium (Thermo Fisher Scientific) supplemented with 10 μM Rho kinase inhibitor Y-27632 (BioGems International, Inc., Westlake Village, CA, USA). When cells reached 80–90% confluency, differentiation was initiated by treating cells with 10 μM CHIR99021 (LC Laboratories, Woburn, MA, USA) in RPMI/B27-I medium composed of RPMI 1640 (Thermo Fisher Scientific) supplemented with B27 supplement minus insulin (Thermo Fisher Scientific). After 24 h, medium was replaced with RPMI/B27-I medium. After 48 h, cells were treated with 5 μM IWP4 Wnt inhibitor (Stemgent, Cambridge, MA, USA) in RPMI/B27-I medium for 48 h. From day 5 of treatment, RPMI/B27-I medium was replaced every other day, and from day 9, RPMI/B27 medium composed of RPMI 1640 supplemented with insulin-containing B27 (Thermo Fisher Scientific) was added. RPMI/B27 medium was replaced every other day until beating CMs were observed. For purification of CMs, cells were treated with glucose-free RPMI (Thermo Fisher Scientific) supplemented with 5 mM sodium DL-lactate (Sigma-Aldrich), 0.211 mg/ml L-ascorbic acid 2-phosphate (Sigma-Aldrich), and 0.5 mg/ml human albumin (Sigma-

Aldrich).

Differentiation of RFP-hiPSCs into CMs utilized the same protocol with slight modifications. Cells were treated with 6 μM CHIR99021 in RPMI/B27-I medium for 48 h, after which medium was replaced with RPMI/B27-I medium. After 24 h, 5 μM IWR-1-endo (Cayman Chemical, Ann Arbor, MI, USA) in RPMI/B27-I medium was added to the cells for 48 h. From day 5 after treatment, RPMI/B27 medium was replaced every other day.

CF differentiation from hiPSCs was conducted as reported[10]. In detail, hiPSCs at 80–90% confluency were cultured in RPMI/B27-I medium containing 6 μM CHIR99021 for 2 days, RPMI/B27-I medium for 1 day, and RPMI/B27-I medium containing 5 μM IWR-1 (Sigma-Aldrich) for 2 days. Differentiated cells were replated on hESC-qualified Matrigel-coated culture plates and treated with 5 μM CHIR99021 and 2 μM retinoic acid (Sigma-Aldrich) in Advanced DMEM/F12 medium (Thermo Fisher Scientific) for 3 days and then cultured in Advanced DMEM/F12 medium without supplements for 4 days. Cells were replated once more and cultured in Fibroblast Growth Medium-3 (PromoCell, Heidelberg, Germany) containing 10 ng/ml FGF2 (R&D Systems) and 10 μM SB431542 (Sigma-Aldrich) for 6 days.

Computational simulation analysis and oxygen level detection

Computational simulations were performed using the Laminar Flow interface and the Transport of Diluted Species interface with COMSOL Multiphysics software (COMSOL Inc., Burlington, MA, USA). Flow in the device system was solved using the Navier-Stokes equation and oxygen transport was determined using both convection and diffusion. The values used for the simulations were based on previously reported studies. Density and dynamic viscosity of the medium were set at 1030 kg/m^3 and 0.0025 Pa·s, respectively, for simulation[11]. In the simulation of oxygen transport, the oxygen concentration in the medium

was set at 222.5 μM and the diffusion coefficient of the oxygen in the medium and in the spheroid was set at 2.4×10^{-5} cm^2/s and 3.0×10^{-6} cm^2/s, respectively, according to previous studies[12, 13]. The oxygen consumption rate of the cardiac tissue was estimated to be 0.45 pmol/cell/h, according to a previous study[14]. The oxygen levels of cardiac tissues were detected using the Image-iT™ red hypoxia reagent (Thermo Fisher Scientific), as used in previous research on cardiac spheroids[13]. Fluorescence images of live cardiac tissues treated with the hypoxia reagent were taken using confocal microscopy, maintaining the same thickness from the bottom of the cardiac tissues. A series of 15 z-slice confocal images were captured at intervals of 10.03 μm and then stacked to generate a z-stack image with a total thickness of 140.42 μm. Then, the relative fluorescence intensity and fluorescence area (%) of cardiac tissues in each group were quantified using ImageJ software (National Institutes of Health, Bethesda, MD, USA).

Histology

Decellularized porcine heart tissues, native porcine heart tissues, HEM hydrogel-injected mouse skin tissues, and cardiac tissue-transplanted rat heart tissues were fixed with 10% formalin solution (Thermo Fisher Scientific) for 12 h, embedded in paraffin blocks, and sectioned to 6-μm (for HEM characterization) or 5-μm (for transplantation experiments) thicknesses. Cardiac tissues produced *in vitro* were fixed with 10% formalin solution for 1 h, embedded in OCT compound (Leica Biosystems, Wetzlar, Germany), and frozen. OCT-embedded frozen samples were sectioned to 8-μm thickness. Slides were stained with H&E, Alcian blue, and Masson's trichrome according to the manufacturer's instructions. Imaging was performed using a VS120-S5 slide scanner (Olympus Corporation, Tokyo, Japan) and a Pannoramic MIDI slide scanner (3DHISTECH, Budapest, Hungary).

Immunostaining

OCT-embedded cardiac tissue sections or fixed whole cardiac tissues (for whole-mount staining) were permeabilized with 0.5% Triton X-100 (Sigma-Aldrich) for 30 min at room temperature. Paraffin-embedded tissue sections, including HEM hydrogel-injected mouse skin tissues and cardiac tissue-transplanted rat heart tissues, were deparaffinized, rehydrated, and permeabilized with Triton X-100. Samples were blocked with 5% bovine serum albumin (MP Biomedicals, Solon, OH, USA) containing 2% horse serum (Thermo Fisher Scientific) for at least 2 h at room temperature. Samples were incubated overnight at 4°C with the following primary antibodies: mouse anti-GATA-4 (#sc-25310, 1:200, Santa Cruz Biotechnology, Dallas, TX, USA), mouse anti-α-actinin (#A7811, 1:400, Sigma-Aldrich), rabbit anti-cardiac troponin T (cTnT, #ab45932, 1:400, Abcam), mouse anti-cardiac troponin T (#MA5-12960, 1:100, Thermo Fisher Scientific), mouse anti-cardiac troponin T (#ab8295, 1:200, Abcam), rabbit anti-cardiac troponin I (cTnI, #ab47003, 1:200, Abcam), rabbit anti-connexin-43 (CX43, #C6219, 1:200, Sigma-Aldrich), rabbit anti-connexin-43 (#ab11370, 1:300, Abcam), mouse anti-CD31 (#BBA7, 10 μg/ml, R&D Systems), rabbit anti-CD31 (#ab28364, 1:200, Abcam), mouse anti-DDR2 (#sc-81707, 1:200, Santa Cruz Biotechnology), mouse anti-vimentin (VIM, #MAB1681, 1:100, Millipore Corporation, Burlington, MA, USA), rabbit anti-collagen type 1 (#234167, 1:200, Millipore Corporation), mouse anti-α-smooth muscle actin (α-SMA, #sc-53142, 1:200, Santa Cruz Biotechnology), rat anti-CD11b (#ab8878, 1:200, Abcam), mouse anti-CD45 (#AF114, 200 μg/ml, R&D Systems), mouse anti-inducible nitric oxide synthase (iNOS, #sc7271, 1:200, Santa Cruz Biotechnology), rabbit anti-CD206 (#ab64693, 1:200, Abcam), mouse anti-CD80 (#MA5-42562, 1:100, Thermo Fisher Scientific), rabbit anti-CD163 (#ab182422, 1:200, Abcam), and rabbit anti-cleaved caspase-3 (#9661S, 1:400, Cell Signaling Technology, Danvers, MA, USA). Samples were washed three times with PBS and incubated with the following species-

specific fluorescence-conjugated secondary antibodies for 24 h at 4°C: anti-mouse Alexa Fluor 488 (#A11001, 1:200, Thermo Fisher Scientific), anti-rabbit Alexa Fluor 488 (#A11008, 1:200, Thermo Fisher Scientific), anti-mouse Alexa Fluor 488 (#A21202, 1:500, Thermo Fisher Scientific), anti-rabbit Alexa Fluor 488 (#A21206, 1:500, Thermo Fisher Scientific), anti-rat Alexa Fluor 488 (#A11006, 1:200, Thermo Fisher Scientific), anti-rabbit Alexa Fluor 555 (#4413S, 1:500, Cell Signaling Technology), anti-mouse Alexa Fluor 594 (#A11005, 1:200, Thermo Fisher Scientific), anti-rabbit Alexa Fluor 594 (#A11012, 1:200, Thermo Fisher Scientific), and anti-rabbit Alexa Fluor 647 (#A31573, 1:500, Thermo Fisher Scientific). Samples were washed with PBS and counterstained with 2-(4-amidinophenyl)indole-6-carboxamidine dihydrochloride (DAPI, 1:500, TCI). F-actin staining was performed using rhodamine phalloidin solution (#R415, 1:400, Thermo Fisher Scientific). T-tubule staining was performed using fluorescein isothiocyanate (FITC)-conjugated WGA (#L4859, 5 μg/ml, Sigma-Aldrich) and denatured collagen staining was performed using collagen hybridizing peptide (CHP, #FLU300, 20 μM, 3HELIX, Salt Lake City, UT, USA). Images of stained samples were acquired using confocal microscopy (LSM900, Carl Zeiss, Jena, Germany).

Quantitative PCR (qPCR) analysis

To investigate gene expression in cardiac tissues, mRNA was extracted from the tissues using an RNeasy Mini Kit (Qiagen, Hilden, Germany). cDNA was prepared using a cDNA synthesis kit (#6110A, TaKaRa). qPCR analysis was performed using TaqMan Fast Universal PCR Master Mix (Thermo Fisher Scientific) on a StepOnePlus Real-Time PCR System (Thermo Fisher Scientific). The following primers for qPCR were used: *NPPA* (Hs00383230_g1), *TNNT2* (Hs00943911_m1), *TNNI1* (Hs00913333_m1), *TNNI3* (Hs00165957_m1), *MYL2* (Hs00166405_m1), *MYL7* (Hs01085598_g1), *MYH6*

(Hs01101425_m1), *MYH7* (Hs01110632_m1), *SCN5A* (Hs00165693_m1), *CACNA1C* (Hs00167681_m1), *PECAM1* (Hs00169777_m1), *CDH5* (Hs00901465_m1), *vWF* (Hs01109446_m1), *COL1A1* (Hs00164004_m1), *PDGFRA* (Hs00998018_m1), *SPARC* (Hs00234160_m1), *SFRP2* (Hs00293258_m1), *FOSL1* (Hs00759776_s1), and *FBN1* (Hs00171191_m1). The expression of each gene was normalized to that of glyceraldehyde 3-phosphate dehydrogenase (*GAPDH*; Hs02786624_g1).

RNA sequencing analysis

RNA was extracted from cardiac tissue samples using an RNeasy Mini Kit (Qiagen) and transferred to DNA Link Inc. (Seoul, Korea) for RNA sequencing. Sequencing libraries were prepared using an Illumina TruSeq Stranded mRNA Library Prep Kit (Illumina, San Diego, CA, USA) according to the manufacturer's instructions. Libraries were pooled and sequenced using a NovaSeq 6000 platform (Illumina). Sequenced reads were mapped to a reference genome (Human hg19) using Tophat (v2.0.13). Differentially expressed genes (DEGs) were calculated using Cuffdiff (v2.2.1). DEGs with a 2-fold change and a false discovery rate (FDR) < 0.1 were selected for Gene Ontology (GO) analysis. Functional GO annotation was performed using the DAVID program (https://david.ncifcrf.gov). A heatmap was created using Cluster 3.0 and Java TreeView 1.2.0. A PCA plot was generated using the ggfortify and ggplot2 packages (version 3.3.5) in Rstudio (version 2022.02.3; RStudio, Boston, MA, USA). A dot plot for GO term visualization was generated using the ggplot2 package in Rstudio.

Functional and ultrastructural analyses

Contractility measurements of cardiac tissues in Figures 3g–i, 5a, b, 6c, d, and 6k and o were performed using contraction videos and the MUSCLEMOTION plugin for ImageJ software[15]. Contraction videos were acquired using an IX71 microscope (Olympus

Corporation) and Ocular image acquisition software (Teledyne Photometrics, Birmingham, UK). Contraction parameters were calculated from the mean values of several peaks from each cardiac tissue and beats per minute (BPM) were calculated from the peak-to-peak time. For electrical pacing experiments, biphasic electrical pulses (3 V, 1 Hz, and 100 msec) optimized for cardiac tissues were applied using a WPG100e electrochemical workstation (WonATech, Seoul, Korea). Calcium transient analysis of cardiac tissues was performed using 5 μM Fluo-4 AM staining (Thermo Fisher Scientific). Cardiac tissues were treated with Fluo-4 AM and incubated for 30 min at 37°C. Fluorescent videos of calcium transients were obtained using an IX71 microscope and Ocular image acquisition software. Cardiac tissue sections and ultrastructures of CMs were observed using field emission-scanning electron microscopy (FE-SEM, Teneo VS, FEI, Hillsboro, OR, USA). Cardiac tissues were sectioned using an ultramicrotome (RMC MTXL; Tucson, AZ, USA).

Contractility assay

For contractility tests, cardiac tissues were treated with 10 μM epinephrine (Sigma-Aldrich) and 10 μM isoproterenol (Sigma-Aldrich). Changes in contraction frequency were analyzed using MUSCLEMOTION. Separately, cardiac tissues were treated with various concentrations of nifedipine (Sigma-Aldrich), and contractility was analyzed using SoftEdge™ Acquisition software (IonOptix, Milton, MA, USA) as shown in Figure 5c and Supplementary Figure 6. Specifically, cardiac tissues were paced on the recording chamber perfused with a physiological Tyrode solution (120 mM NaCl, 5.4 mM KCl, 5 mM $MgSO_4$, 0.2 mM $CaCl_2$, 5 mM Na-pyruvate, 5.5 mM glucose, 20 mM taurine, 10 mM HEPES, and 29 mM mannitol, pH 7.4, NaOH) at 3 ml/min. Electrical field stimuli were applied using an electric stimulator (MyoPacer, IonOptix), and only tissues that beat stably were used in the drug tests. Contraction-relaxation waves were monitored and recorded digitally using

SoftEdge™ Acquisition software, which detects motion at the boundary of cardiac tissues. All experiments were conducted at 36 ± 1°C. Waveforms were collected and analyzed at baseline (control) and at each testing concentration of nifedipine (a negative inotrope). The waveforms from at least 10 steady-state contractions were averaged for the control and each dose of nifedipine. Three parameters for cardiac contractility, including peak shortening, contraction velocity, and relaxation velocity, were analyzed.

Multi-electrode array (MEA) assay

Cardiac tissues were treated with various concentrations of E-4031 (Sigma-Aldrich) and nifedipine, and electrophysiological changes in response to the drugs were detected using MEA assays. Eight drugs with known clinical Torsades de Pointes (TdP) risks were evaluated, including seven drugs (vandetanib, bepridil, quinidine, chlorpromazine, terfenadine, clarithromycin, and diltiazem) purchased from Sigma-Aldrich and one drug (ranolazine) purchased from USP (Rockville, MD, USA). For all test drugs, 1000× stock solutions were prepared for the highest test concentration in dimethyl sulfoxide (DMSO). Stock solutions were serially diluted in DMSO. MEA assays were performed using a Maestro MEA system with 12-well MEA plates (Axion BioSystems Inc., Atlanta, GA, USA). Multi-well MEA plates were pre-coated with fibronectin (50 μg/mg) for 30 min in a 5% CO_2 incubator at 37°C. Cardiac tissues were plated on the MEA plates and allowed to attach to the wells for 30 min at 37°C. Cardiac tissues were maintained on MEA plates for 5–7 days with a half-volume medium change every 2–3 days. On the day of assessment, the medium was fully replaced at least 4 h before the recordings to allow for equilibration and correction of medium volume.

After docking the MEA plate containing the cardiac tissues on the MEA equipment, the device automatically adjusted and maintained the temperature at 37°C and CO_2 level at

5%. Baseline levels were recorded for 1 min at 5-min intervals for a total of 30 min. The experimental protocol employed an accumulative treatment, which consisted of the administration of four serial concentrations of each test drug. During drug testing, MEA signals were recorded for 1 min at 5-min intervals for a total of 20 min for each dose. Drug administration was performed by gentle manual pipetting. A volume of 100 μl of medium was removed from the MEA plate and mixed with 1 μl of stock solution for each dose and gently returned to the MEA plate along the edge of the well. Data from the last 1-min recording of each drug dose were analyzed offline using AxIS software (version 2.3.2.4, Axion BioSystems Inc.). Three major parameters were derived from the cardiac field potential under baseline (control) and post-dose conditions, including BPM, field potential amplitude (FPA), and FPD_{cF}, which represented the field potential duration corrected heart rate using Fridericia's formula[16].

To assess the electrophysiological characteristics of our LQTS cardiac tissues, an MEA system (Tucker-Davis Technologies Inc, Alachua, FL, USA) was used, which consisted of RZ2 amplifier processor, PZ5 Neurodigitizer, MZ60 MEA interface, and a computer with Synapse program. The recordings were performed at a sampling rate of 24,414 Hz with 60 Hz notch filter and a 0.1–300 Hz bandpass filter.

Evaluation of heart function by echocardiography

Echocardiography was used for functional evaluation of ischemia/reperfusion-injured hearts. Rats were lightly anesthetized with 2% isoflurane. Data were recorded using a transthoracic echocardiography system equipped with a 15-MHz L15-7io linear transducer (Affiniti50G; Philips, Andover, MA, USA). Base echocardiography was performed 1 week after ischemia/reperfusion modeling, and additional echocardiography was performed 1, 2, 4, and 6 weeks after cardiac tissue transplantation. The echocardiography operator was blinded to

the group allocation during the experiment. Ejection fraction (EF) and fractional shortening (FS), which are indexes of left ventricular (LV) systolic function, were calculated with the values of left ventricular end-diastolic volume (LVEDV), left ventricular end-systolic volume (LVESV), left ventricular end-diastolic diameter (LVEDD), and left ventricular end-systolic diameter (LVESD) using the following equations, respectively:

EF (%) = [(LVEDV-LVESV)/LVEDV] × 100

FS (%) = [(LVEDD-LVESD)/LVEDD] × 100

Hemodynamic measurements

Hemodynamic measurements were performed before euthanasia at 4 weeks post-transplantation. Rats were lightly anesthetized with 2% isoflurane, and thoracotomy was performed without bleeding. The LV apex of the heart was pierced with a 26-gauge needle and a 2F conductance catheter (SPR-838; Millar Instruments, Houston, TX, USA) was placed in the LV. The pressure-volume (PV) parameters were uninterruptedly recorded using a PV conductance system (MPVS Ultra; emka TECHNOLOGIES, Paris, France) coupled with a digital converter (PowerLab 16/35; ADInstruments, Chalgrove, UK). Cardiac output, stroke volume, maximal rate of pressure changes during systole (dP/dt_{max}), minimal rate of pressure changes during diastole (dP/dt_{min}), and maximum volume at end-diastole (V_{max}) were quantified using recorded values. Load-independent measurements of cardiac function, including the slopes of the end-systolic pressure-volume relationship (ESPVR) and end-diastolic pressure-volume relationship (EDPVR), were achieved with different preloads, which were elicited by inferior vena cava (IVC) occlusion using a needle holder. Hypertonic saline (50 μl of 20% NaCl) was injected into the left jugular vein to evaluate parallel conductance after hemodynamic measurements. For calibrating the data, blood was collected from the LV apex using heparinized syringes and placed into cuvettes to convert the

conductance signal to volume using the catheter. The absolute blood volume of rat hearts was confirmed by calibrating the parallel conductance and the cuvette conductance.

Electrocardiogram (ECG) analysis of transplanted rat heart

ECG analysis was performed 6 weeks after transplantation using a Langendorff isolated heart perfusion system. Rats were anesthetized using 2% isoflurane (Hana Pharm, Seongnam, Korea), and their hearts were rapidly excised and mounted on an aortic cannular. Then, the hearts were perfused with oxygenated (95% O_2 + 5% CO_2) modified Krebs-Henseleit buffer (pH 7.4) containing 11.1 mM glucose, 118 mM NaCl, 4.69 mM KCl, 2.0 mM $CaCl_2$, 1.17 mM $MgSO_4$, 1.18 mM KH_2PO_4, and 25 mM $NaHCO_3$, and maintained at 37°C. The hearts were perfused at a constant flow rate of 15 ml/min, and ECG electrodes were attached to the epicardium. Data were recorded using the PowerLab system (ADInstruments). Following a 20-minute stabilization period, ECG measurements were taken over a 10-minute duration.

Quantitative image analysis of the myocardial infarction model

To visualize the fibrosis area and viable myocardium in the injured hearts, paraffin-embedded heart tissues were sectioned and stained with Masson's trichrome. The fibrotic region (blue) and viable myocardium (red) areas were quantified manually using ImageJ software. Capillary density, engrafted hiPSC-CM, and lateralized CX43 were quantified using the count values from immunofluorescent images of $CD31^+$ capillary, RFP^+ human $cTnT^+$ cells, and lateralized $CX43^+$ junctions, respectively. The number of total CMs was calculated as the number of $cTnT^+$ cells, and the denatured collagen area was quantified by measuring the CHP^+ region. Quantification of immunofluorescent images was conducted in three randomly selected microscopic fields (/mm^2) per tissue.

Supplementary Figures

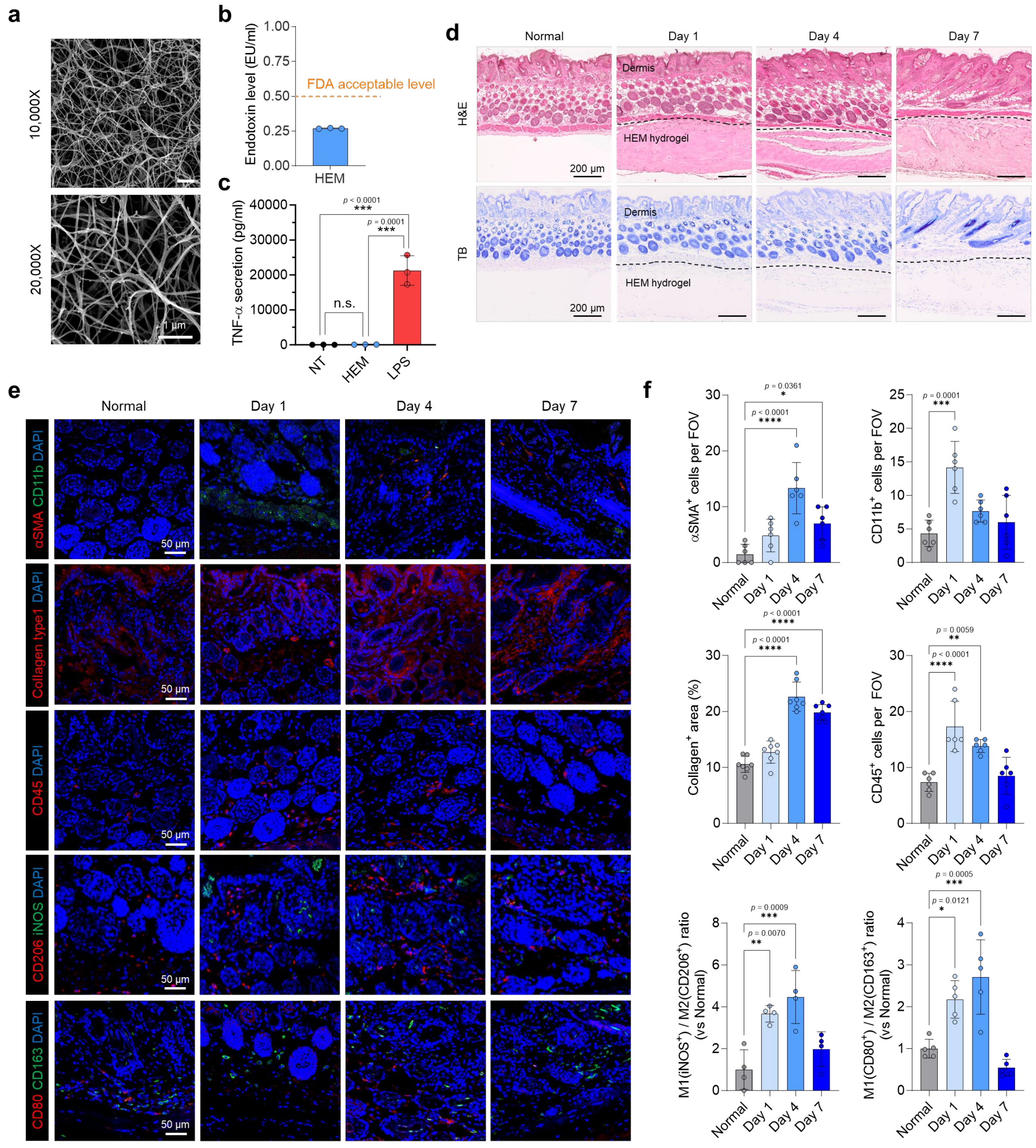

Supplementary Figure 1. Fabrication of heart extracellular matrix (HEM) hydrogel and biocompatibility evaluation *in vitro* and *in vivo*. (**a**) Scanning electron microscopy images showing internal ultrastructures of HEM hydrogels (scale bars = 1 μm). (**b**) Endotoxin levels detected in HEM hydrogels. The yellow dotted line indicates the acceptable level according to the Food and Drug Administration for medical devices in humans. (**c**) Tumor necrosis factor-α (TNF-α) secretion levels by RAW 264.7 macrophages cultured with HEM hydrogels for 6 h ($N = 3$, biological replicates, $***p < 0.001$). Macrophages treated with 1 μg/ml

lipopolysaccharide (LPS) were used for control. (**d**) Hematoxylin and eosin (H&E) staining and toluidine blue (TB) staining of skin tissues from mice that received a subcutaneous injection of HEM hydrogels (scale bars = 200 μm). (**e**) Immunofluorescent images of activated fibroblasts (α-SMA and Collagen type 1), immune cells (CD11b and CD45), M1 macrophages (iNOS and CD80), and M2 macrophages (CD206 and CD163) at the subcutaneous injection site of mice 1, 4, and 7 days after HEM hydrogel injection (scale bars = 50 μm, $N = 4$ for iNOS/CD206 images, $N = 5$ for CD80/CD163 images, $N = 6$ for α-SMA/CD11b and CD45 images, and $N = 7$ for collagen type 1 images, biological replicates). (**f**) Quantification of α-SMA^+, $CD11b^+$, $CD45^+$ cell counts per field of view (FOV), $collagen^+$ area, and M1/M2 ratio ($iNOS^+/CD206^+$ and $CD80^+/CD163^+$) in subcutaneous injection site ($N = 4$ for $iNOS^+/CD206^+$, $N = 5$ for $CD80^+/CD163^+$, $N = 6$ for α-SMA^+, $CD11b^+$, $CD45^+$ cell counts, and $N = 7$ for $collagen^+$ area, biological replicates, $*p < 0.05$, $**p < 0.01$, $***p < 0.001$, and $****p < 0.0001$ versus Normal). Data are presented as means ± S.D. Statistical significance was determined using one-way ANOVA with Tukey's multiple comparisons tests. Non-significant statistical differences are indicated as n.s. ($p > 0.05$).

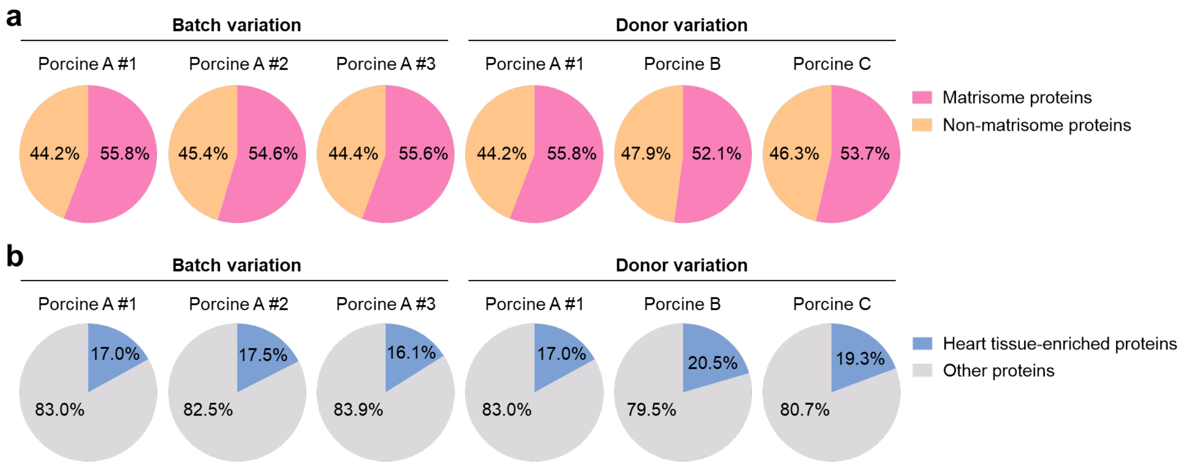

Supplementary Figure 2. Proteomic analysis of variation between different batches and donors of porcine heart extracellular matrix (HEM). (**a**) Relative ratio of matrisome proteins and non-matrisome proteins, and (**b**) percentage of heart tissue-enriched proteins (4-fold higher than other tissues) among the total proteins in porcine HEM samples derived from different batches (#1, #2, and #3 from donor A) and donors (A, B, and C) ($N = 3$, biological replicates).

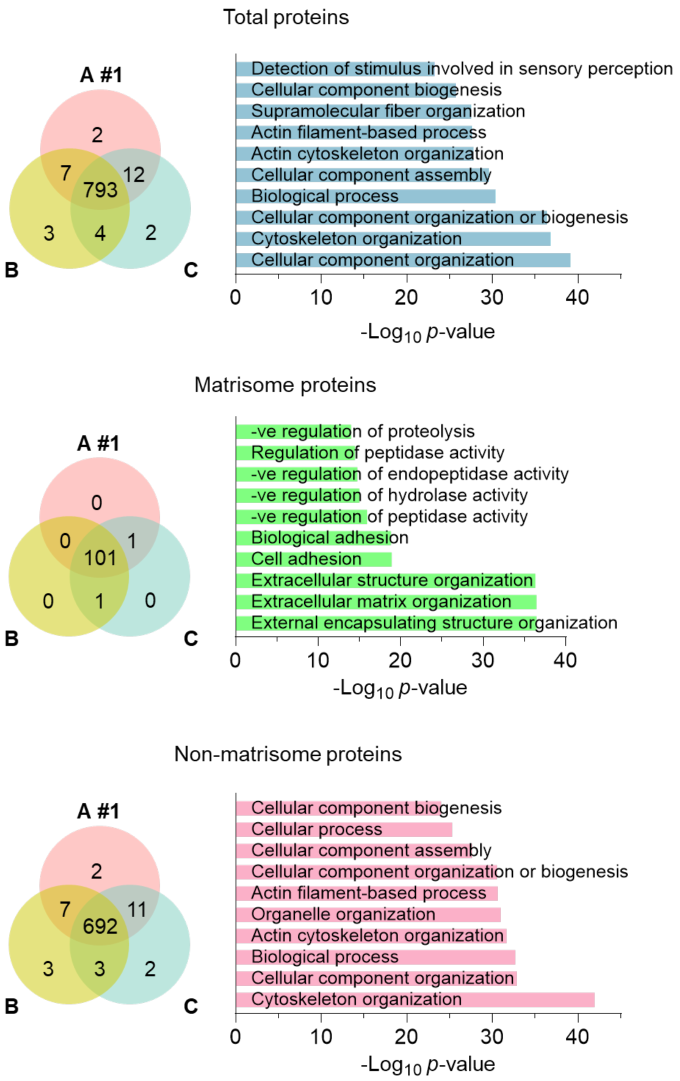

Supplementary Figure 3. Proteomic analysis to identify overlapped proteins (total proteins, matrisome proteins, and non-matrisome proteins) in porcine heart extracellular matrix (HEM) samples derived from different donors (A, B, and C) and Gene Ontology Biological Process (GOBP) analysis ($N = 3$, biological replicates).

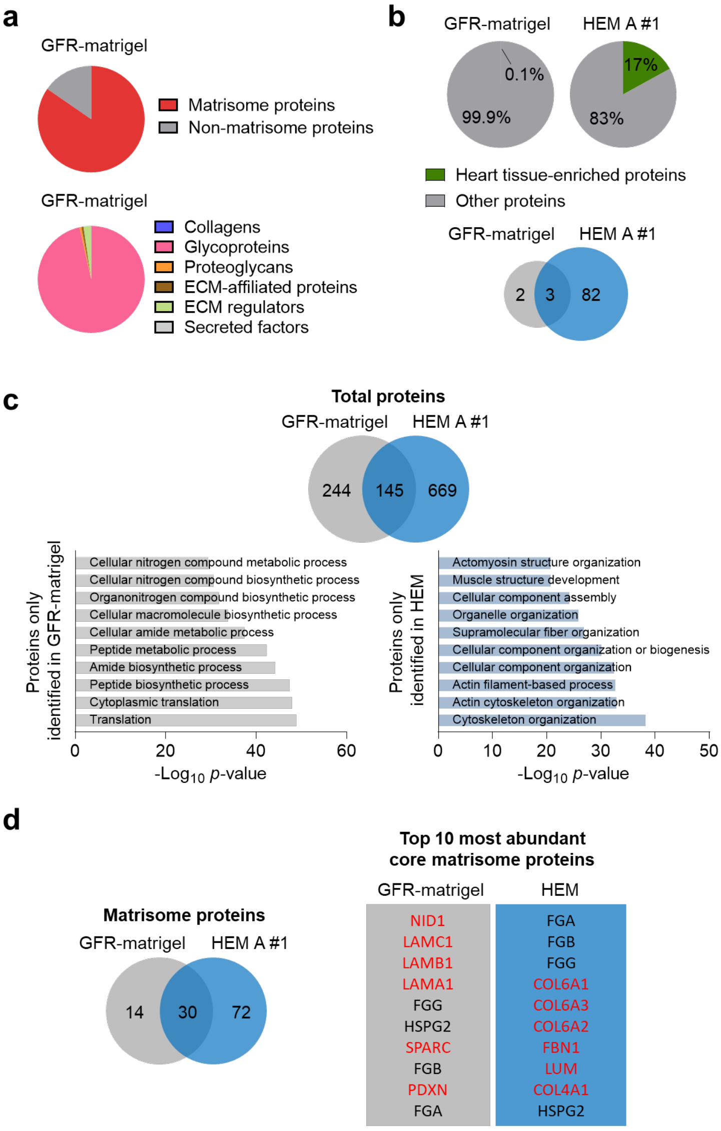

Supplementary Figure 4. Proteomic analysis comparing proteins in porcine heart extracellular matrix (HEM) with those in growth factor reduced Matrigel (GFR-Matrigel). (**a**) Ratios of matrisome proteins and non-matrisome proteins in GFR-Matrigel and extracellular matrix (ECM) compositions in GFR-Matrigel. (**b**) Portion and number of heart tissue-enriched proteins (4-fold higher than other tissues) among total proteins contained in GFR-Matrigel and HEM. (**c**) Number of total proteins contained in GFR-Matrigel and HEM and Gene Ontology Biological Process (GOBP) analysis of proteins only identified in GFR-Matrigel or HEM. (**d**) Number of matrisome proteins and 10 most abundant matrisome proteins in GFR-Matrigel and HEM ($N = 1$ for GFR-Matrigel and $N = 3$ for HEM, biological replicates). The mean value of three biological replicates was used for HEM.

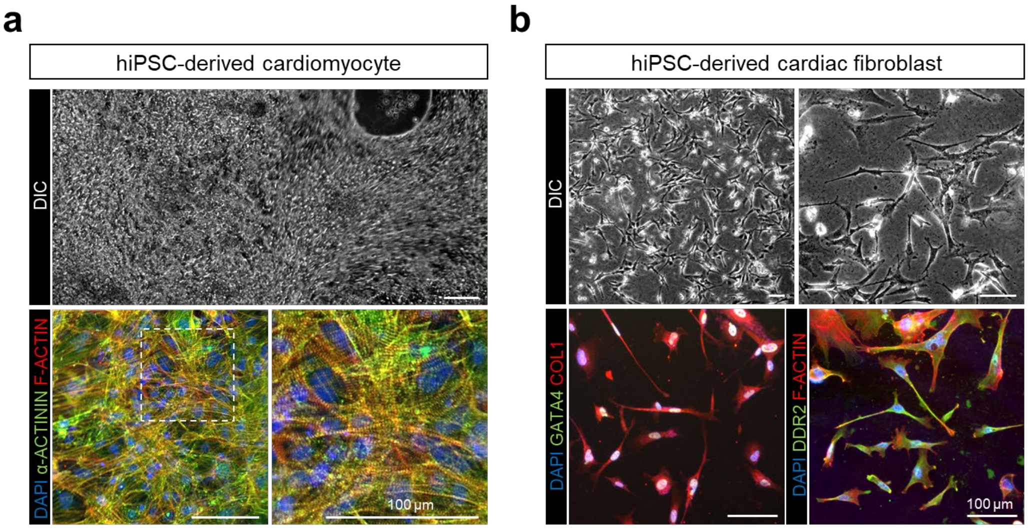

Supplementary Figure 5. Differentiation of cardiomyocytes (CMs) and cardiac fibroblasts (CFs) from human induced pluripotent stem cells (hiPSCs). (**a**) Bright-field images of CMs differentiated from hiPSCs for 14 days and immunofluorescent images of α-actinin and F-actin in CMs (scale bars = 100 μm). (**b**) Bright-field images of CFs differentiated from hiPSCs for 22 days and immunofluorescent images of GATA4, COL1, DDR2, and F-actin in CFs (scale bars = 100 μm).

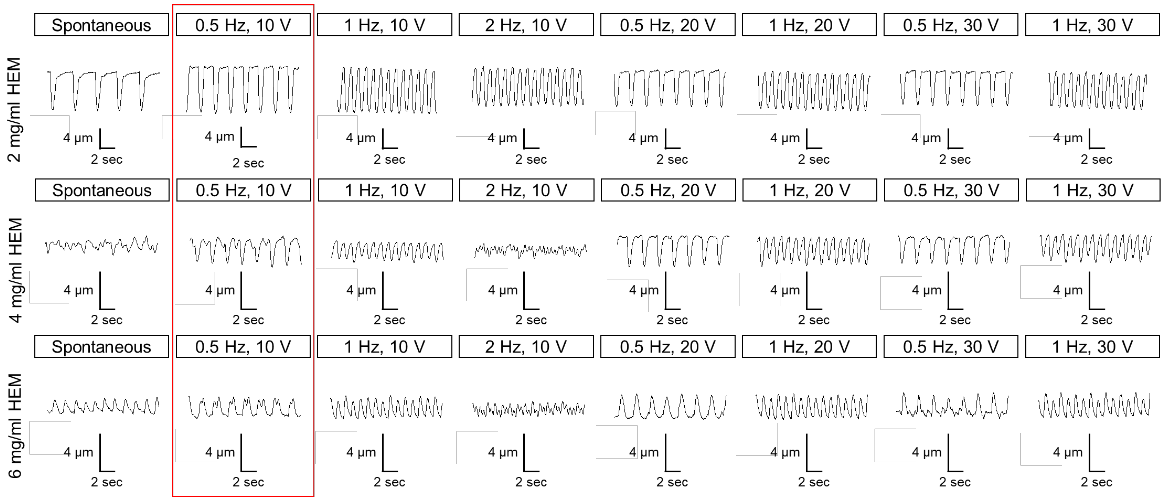

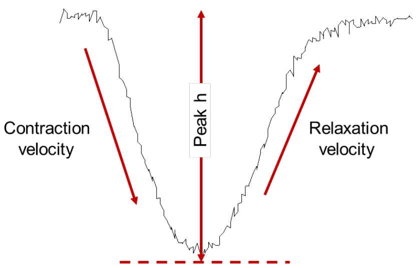

Parameters	HEM concentration		
	2 mg/ml (*N* = 3)	4 mg/ml (*N* = 3)	6 mg/ml (*N* = 3)
Beating condition at **10 V, 0.5 Hz**	regular	irregular	irregular
Peak h (μm)	6.14 ± 0.04	irregular	irregular
Dep v (μm/ms)	-70.80 ± 9.12	irregular	irregular
Ret v (μm/ms)	14.99 ± 0.13	irregular	irregular
Time to peak 50%	0.09 ± 0.00	irregular	irregular
Time to baseline 50%	0.83 ± 0.00	irregular	irregular
Area of contraction	1.56 ± 0.02	irregular	irregular
Area of relaxation	3.03 ± 0.08	irregular	irregular

Supplementary Figure 6. Contractility of human cardiac tissues fabricated with different concentrations of heart extracellular matrix (HEM) hydrogels. Cardiac tissues prepared with human induced pluripotent stem cell (hiPSC)-derived cardiomyocytes (CMs), cardiac fibroblasts (CFs), and human umbilical cord vein endothelial cells (HUVECs) were cultured for 7–14 days before analysis. Contraction traces and contraction parameters for each cardiac tissue electrically paced with various voltages and frequencies ($N = 3$, biological replicates). The red box indicates the optimal condition of electrical stimulation (10 V and 0.5 Hz) to detect differences between groups. Contraction analysis was performed using SoftEdge™ Acquisition software.

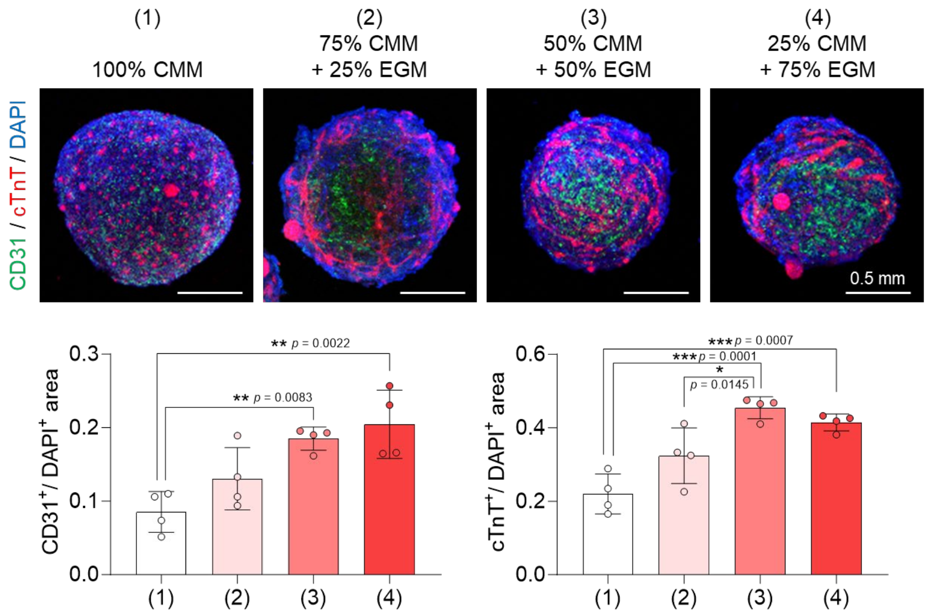

Supplementary Figure 7. Screening of co-culture conditions for human cardiac tissues composed of cardiomyocytes (CMs), endothelial cells (EC), and cardiac fibroblasts (CFs). Immunofluorescent images and quantification of CM (cTnT) and EC (CD31) markers in cardiac tissues cultured in medium at different ratios of CM medium (CMM) and EC medium (EGM) (scale bars = 0.5 mm, $N = 4$, biological replicates, $^{*}p < 0.05$, $^{**}p < 0.01$, and $^{***}p < 0.001$). Cardiac tissues were cultured for 7 days. Data are presented as means ± S.D. Statistical significance was determined using one-way ANOVA with Tukey's multiple comparisons tests.

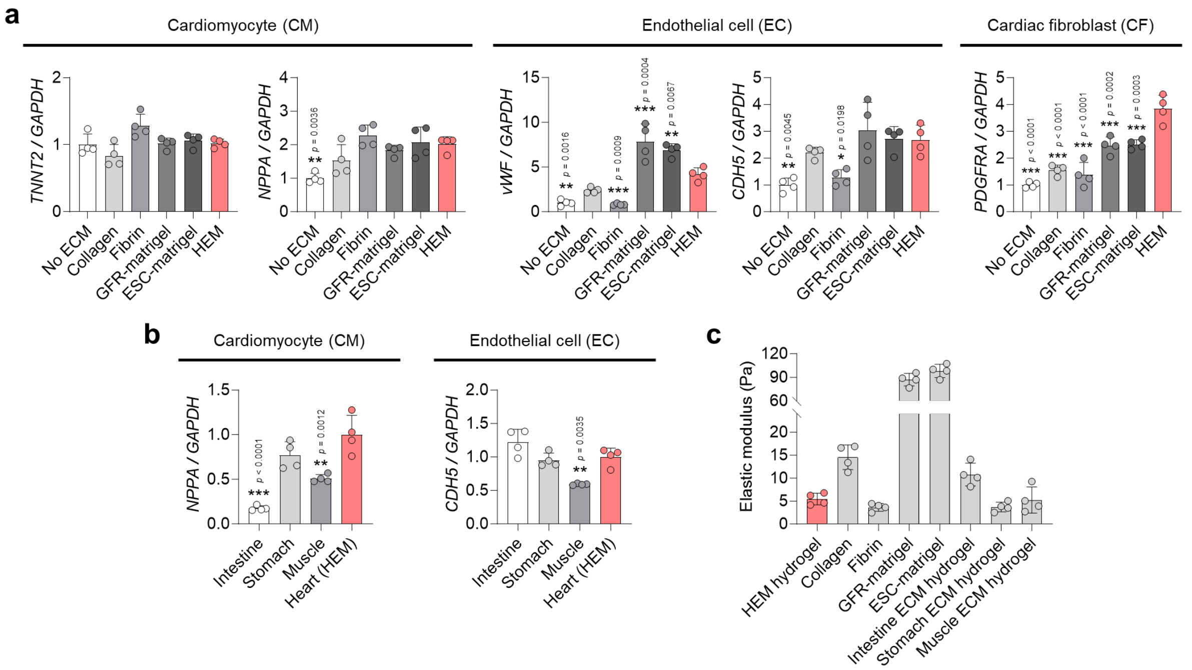

Supplementary Figure 8. mRNA expression levels in human cardiac tissues fabricated using heart extracellular matrix (HEM) hydrogel or other hydrogels. (**a**) Quantitative PCR analysis of relative mRNA expression levels of CM (*TNNT2* and *NPPA*), EC (*vWF* and *CDH5*), and CF (*PDGFRA*) markers in each group of cardiac tissues ($N = 4$, biological replicates, *$p < 0.05$, **$p < 0.01$, and ***$p < 0.001$ versus HEM group). Conventional hydrogels were used as control matrices for cardiac tissue culture. (**b**) Quantitative PCR analysis of relative mRNA expression levels of CM (*NPPA*) and EC (*CDH5*) markers in each group of cardiac tissues ($N = 4$, biological replicates, **$p < 0.01$ and ***$p < 0.001$ versus HEM group). Extracellular matrix (ECM) hydrogels from other decellularized tissues were used as control matrices for cardiac tissue culture. Cardiac tissues were cultured for 7 days in (**a**) and (**b**). (**c**) Elastic moduli of HEM hydrogel, conventional hydrogels, and ECM hydrogels from other decellularized tissues ($N = 4$, biological replicates). Data are presented as means ± S.D. Statistical significance was determined using one-way ANOVA with Tukey's multiple comparisons tests.

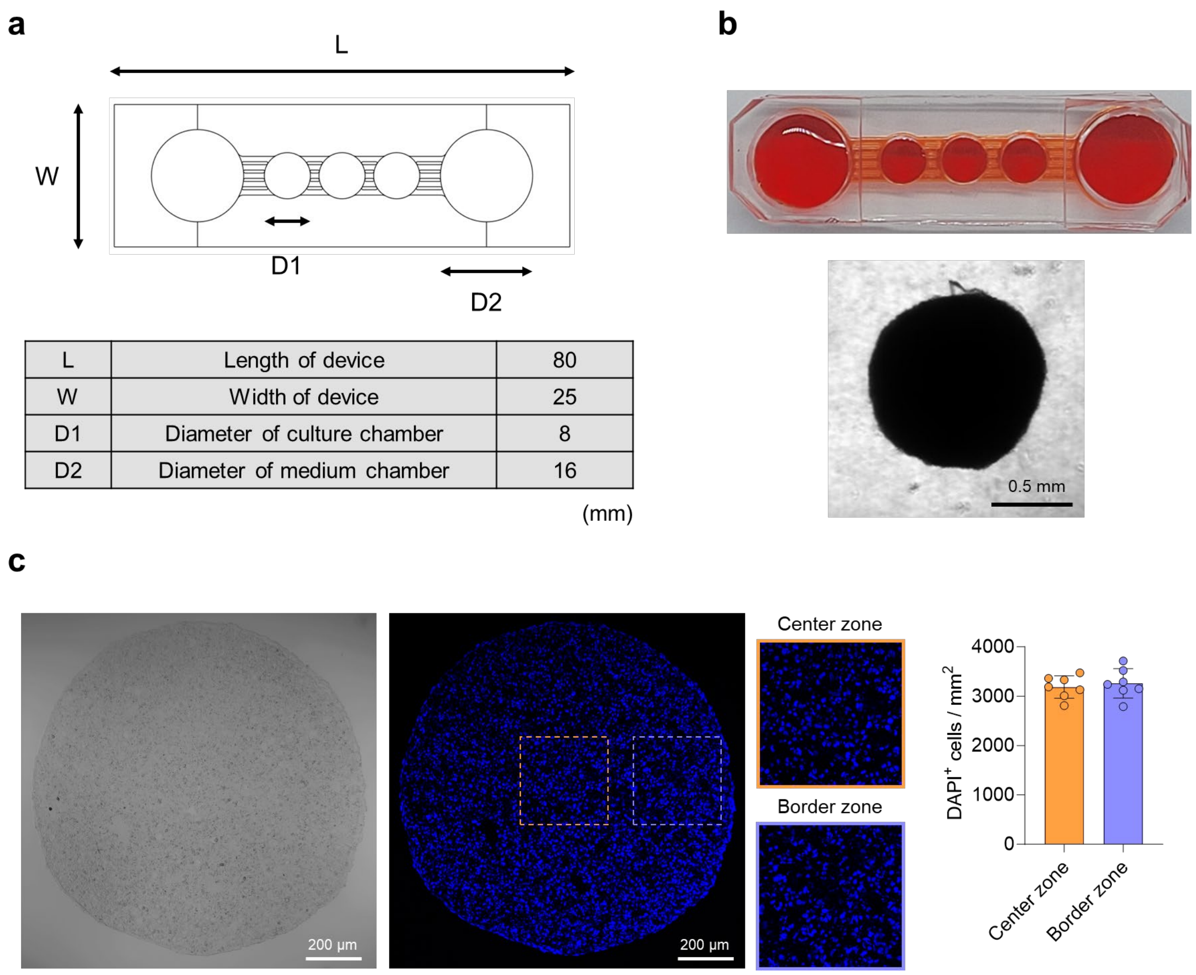

Supplementary Figure 9. Fabrication of microfluidic chips for human cardiac tissue culture and cell density across the diameter of human cardiac tissues. (**a**) Schematic of the microfluidic chip indicating the dimensions, culture chamber, and medium chamber. (**b**) Photograph of the microfluidic chip and bright-field image of cardiac tissue in the culture chamber (scale bar = 0.5 mm). (**c**) Bright-field and DAPI staining images of cardiac tissue section 1 day after the fabrication with heart extracellular matrix (HEM) hydrogel. $DAPI^+$ cells in center zone and border zones within cardiac tissues were quantified (scale bars = 200 µm, $N = 7$, biological replicates). Data are presented as means ± S.D.

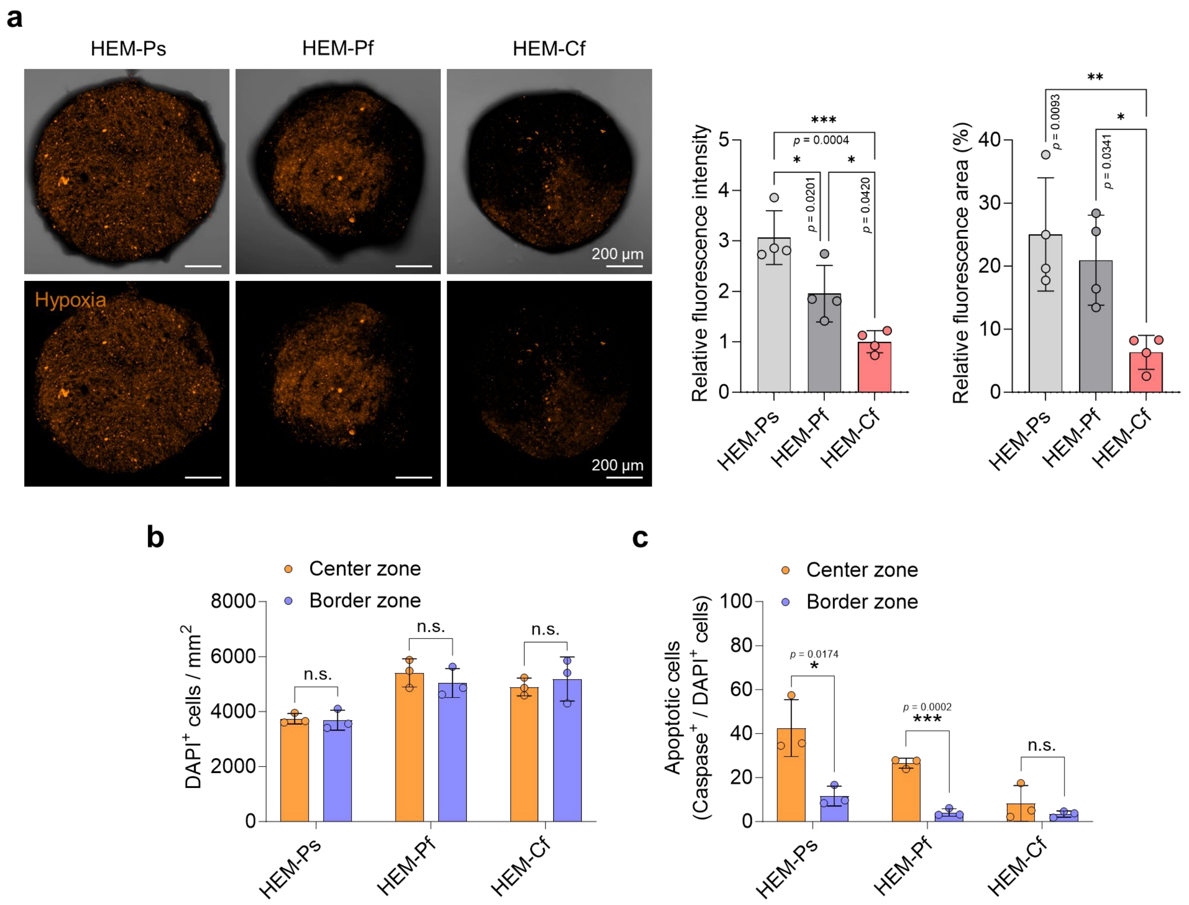

Supplementary Figure 10. Comparison of oxygen levels and apoptotic cell density in human cardiac tissues. (**a**) Fluorescent images of live cardiac tissues stained with Image-iT™ red hypoxia reagent and quantification of relative fluorescence intensity and area. The cardiac tissues were fabricated using heart extracellular matrix (HEM) hydrogel and then cultured in 24-well plates without flow (HEM-Ps; Plate static), 24-well plates with flow (HEM-Pf; Plate flow), and microfluidic chips with flow (HEM-Cf; Chip flow) for 14 days (scale bars = 200 μm, $N = 4$, biological replicates, $*p < 0.05$, $**p < 0.01$, and $***p < 0.001$). (**b**) Quantification of $DAPI^+$ cells and (**c**) apoptotic cells ($Caspase3^+/DAPI^+$) in center zone and border zones within cardiac tissues of each group ($N = 3$, biological replicates, $*p < 0.05$ and $***p < 0.001$). Immunofluorescent images of Figure 3d were used for quantification in (**b**) and (**c**). Data are presented as means ± S.D. Statistical significance was determined using one-way ANOVA with Tukey's multiple comparisons tests in (**a**) and unpaired two-sided Student's *t*-tests in (**b**) and (**c**). Non-significant statistical differences are indicated as n.s. ($p > 0.05$).

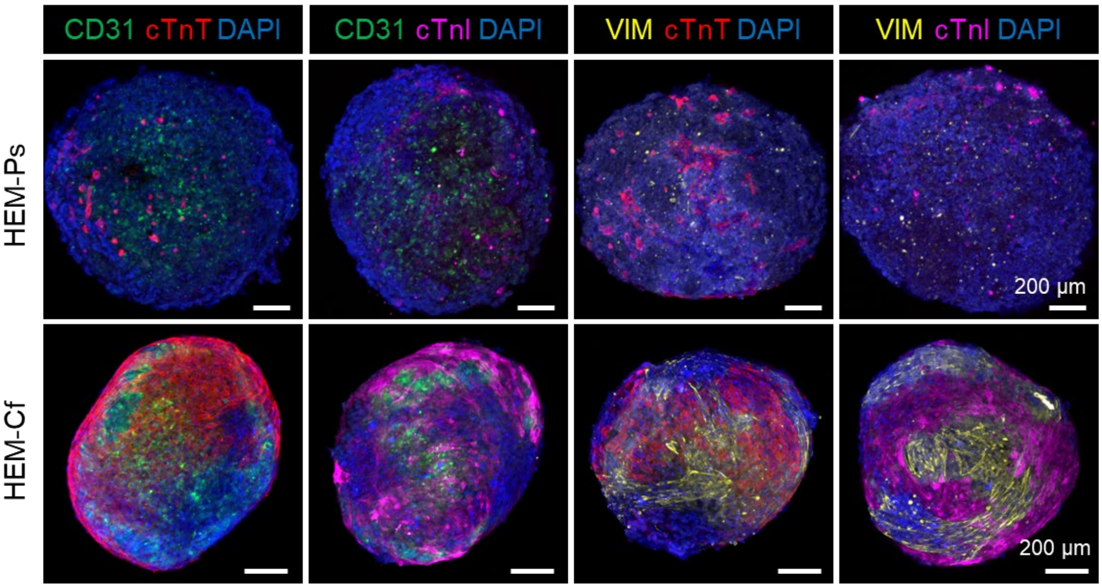

Supplementary Figure 11. Overall protein expression in human cardiac tissues fabricated with heart extracellular matrix (HEM) hydrogel and cultured in well plates without flow (HEM-Ps) and in microfluidic chips with flow (HEM-Cf). Immunofluorescent images of cardiomyocyte (CM; cTnT and cTnI), EC (CD31), and CF (VIM) markers in each group of cardiac tissues cultured for 14 days (scale bars = 200 µm).

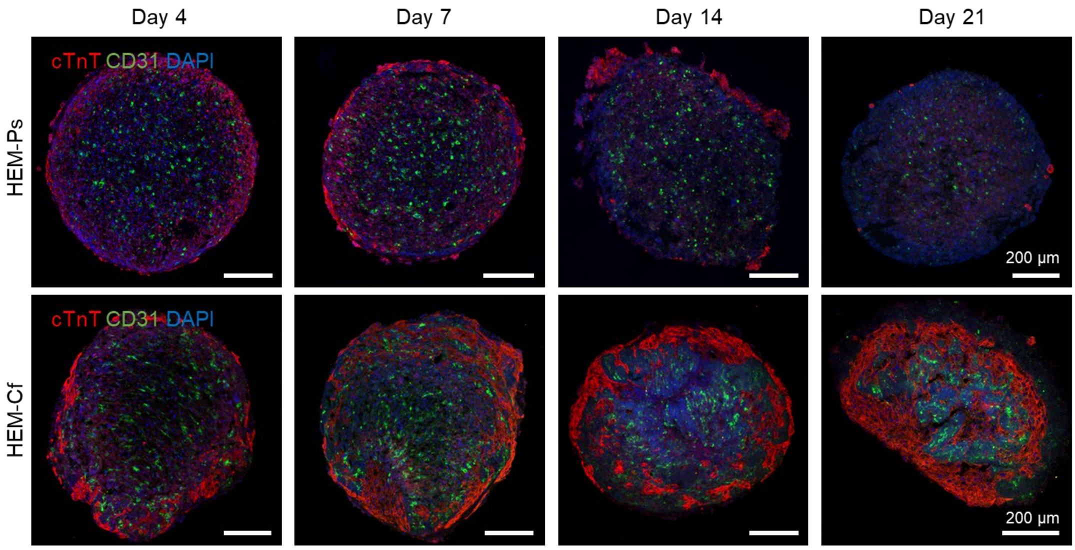

Supplementary Figure 12. Protein expression in the section of human cardiac tissues fabricated with heart extracellular matrix (HEM) hydrogel and cultured in well plates without flow (HEM-Ps) and in microfluidic chips with flow (HEM-Cf). Immunofluorescent images of cardiomyocyte (CM; cTnT) and EC (CD31) markers in the section of each group of cardiac tissues cultured for 4, 7, 14, and 21 days (scale bars = 200 µm).

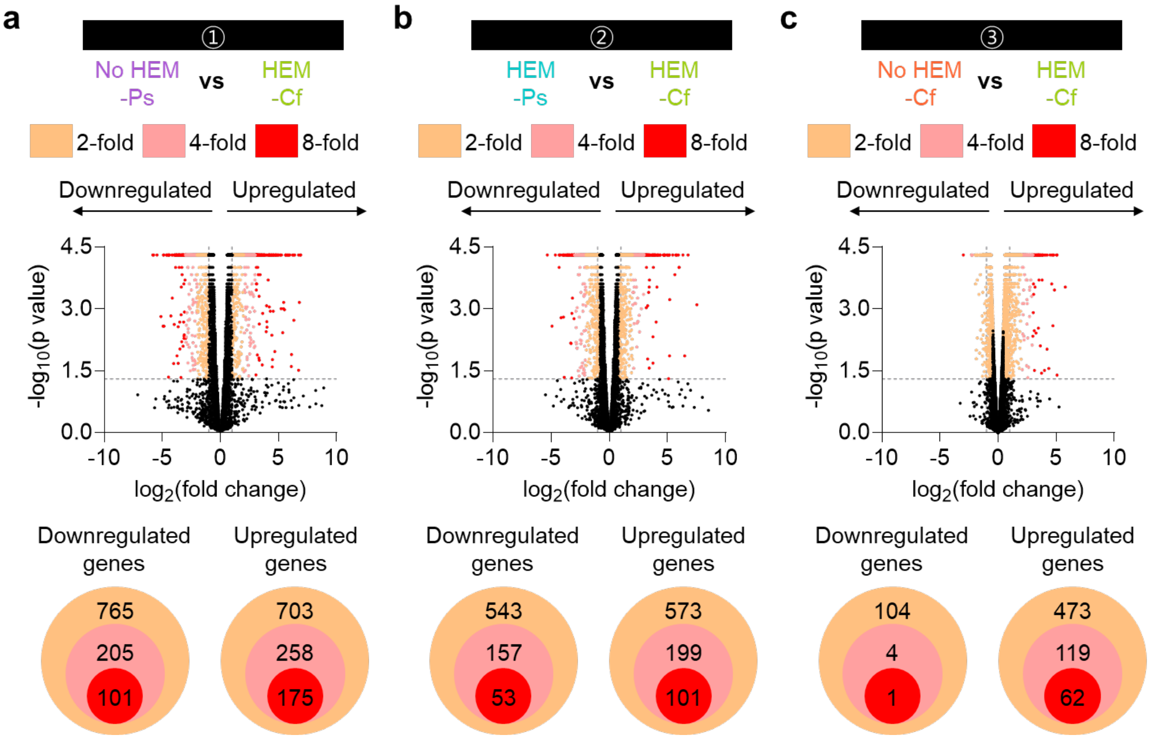

Supplementary Figure 13. Transcriptomic analysis of differentially expressed genes (DEGs) in human cardiac tissues fabricated with heart extracellular matrix (HEM) hydrogel and cultured in microfluidic chip with flow (HEM-Cf) compared to other groups. (**a**, **b**, **c**) Volcano plots displaying genes upregulated or downregulated in the HEM-Cf group as compared to the cardiac tissues fabricated with U-bottom plate and cultured in 24-well plate under static condition (① No HEM-Ps group), cardiac tissues fabricated with HEM hydrogel and cultured in 24-well plate under static condition (② HEM-Ps group), and cardiac tissues fabricated with U-bottom plate and cultured in microfluidic chip with flow (③ No HEM-Cf group). Venn diagram illustrating the number of upregulated and downregulated genes according to fold changes. Cardiac tissues prepared with human induced pluripotent stem cell (hiPSC)-derived cardiomyocytes (CMs), cardiac fibroblasts (CFs), and human umbilical cord vein endothelial cells (HUVECs) were cultured for 14 days.

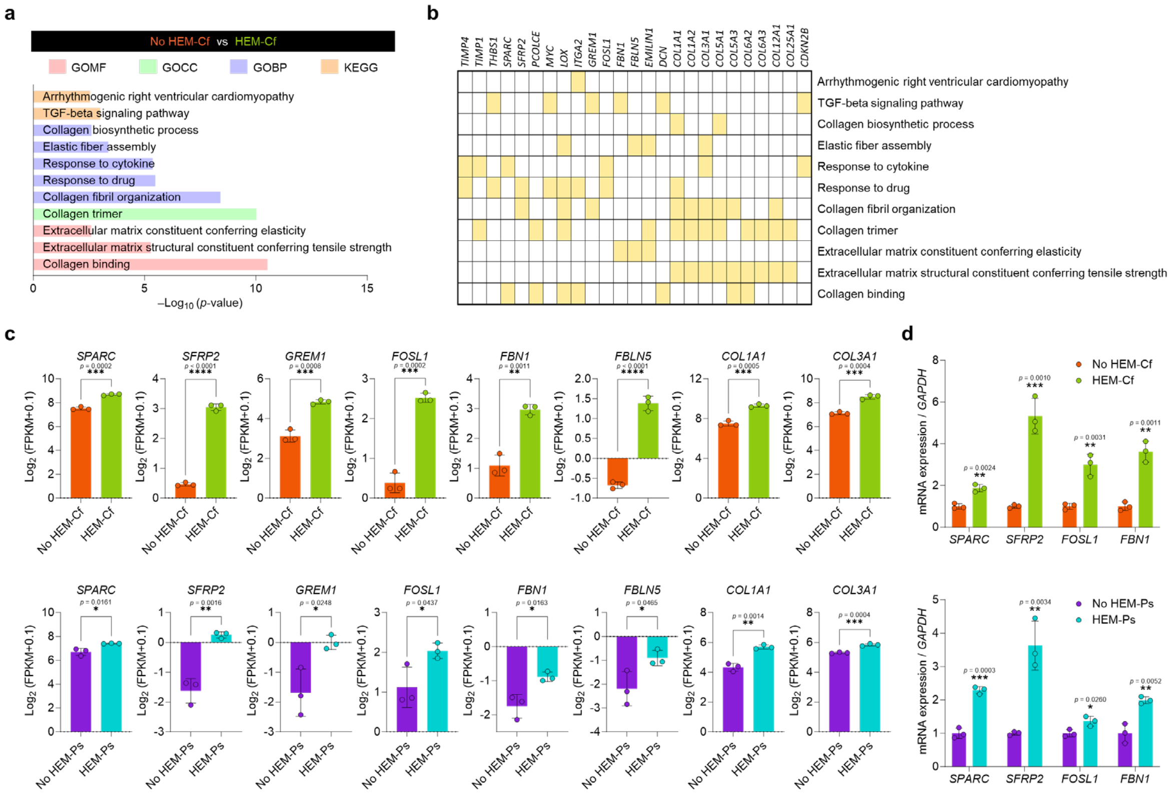

Supplementary Figure 14. The upregulated expression of genes related to drug testing and disease modeling by heart extracellular matrix (HEM) hydrogel. (**a**) Gene Ontology (GO) analysis of differentially expressed genes (DEGs) upregulated in cardiac tissues fabricated using HEM hydrogel and cultured in chips with flow (HEM-Cf) compared to cardiac tissues fabricated using U-bottom plates and cultured in chips with flow (No HEM-Cf). The selected 11 GO terms related to drug testing and disease modeling are displayed. (**b**) The list of DEGs upregulated in the HEM-Cf group compared to the No HEM-Cf group. DEGs overlapping with more than one GO term are shown and GO-annotated DEGs are marked in yellow. (**c**) The expression values (Log_2 [FPKM + 0.1]; FPKM, fragments per kilobase of transcript per million mapped reads) of eight genes (*SPARC*, *SFRP2*, *GREM1*, *FOSL1*, *FBN1*, *FBLN5*, *COL1A1*, and *COL3A1*) in four groups. Four groups (HEM-Cf, No HEM-Cf, HEM-Ps, and No HEM-Ps) were generated according to the fabrication and culture methods, and transcriptomic profiles were compared after 14 days of culture ($N = 3$, biological replicates, $*p < 0.05$, $**p < 0.01$, $***p < 0.001$, and $****p < 0.0001$). (**d**) Quantitative PCR analysis of relative mRNA expression levels of *SPARC*, *SFRP2*, *FOSL1*, and *FBN1* in each group of cardiac tissues ($N = 3$, biological replicates, $*p < 0.05$, $**p < 0.01$, and $***p < 0.001$). Data are presented as means ± S.D. Statistical significance was determined using unpaired two-sided Student's *t*-tests.

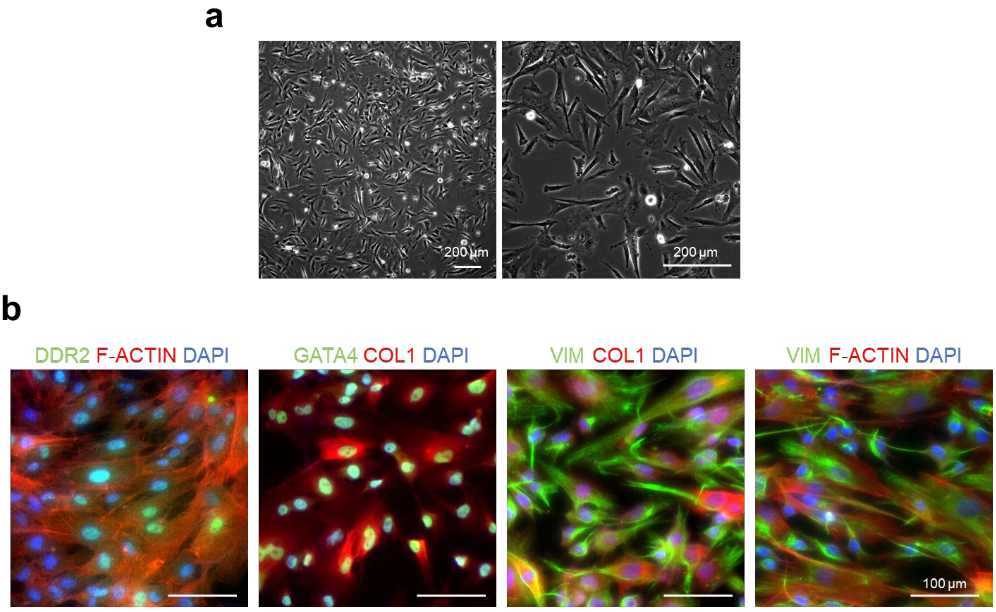

Supplementary Figure 15. Differentiation toward cardiac fibroblasts (CFs) from LQT2 patient induced pluripotent stem cells (iPSCs). (**a**) Bright-field images of LQT2-CFs (scale bars = 200 μm). (**b**) Immunofluorescent images of DDR2, GATA4, COL1, VIM, and F-actin in LQT2-CFs (scale bars = 100 μm). LQT2-CFs were cryopreserved after full differentiation on day 22. Images were obtained using LQT2-CFs cultured over three passages post-cryopreservation.

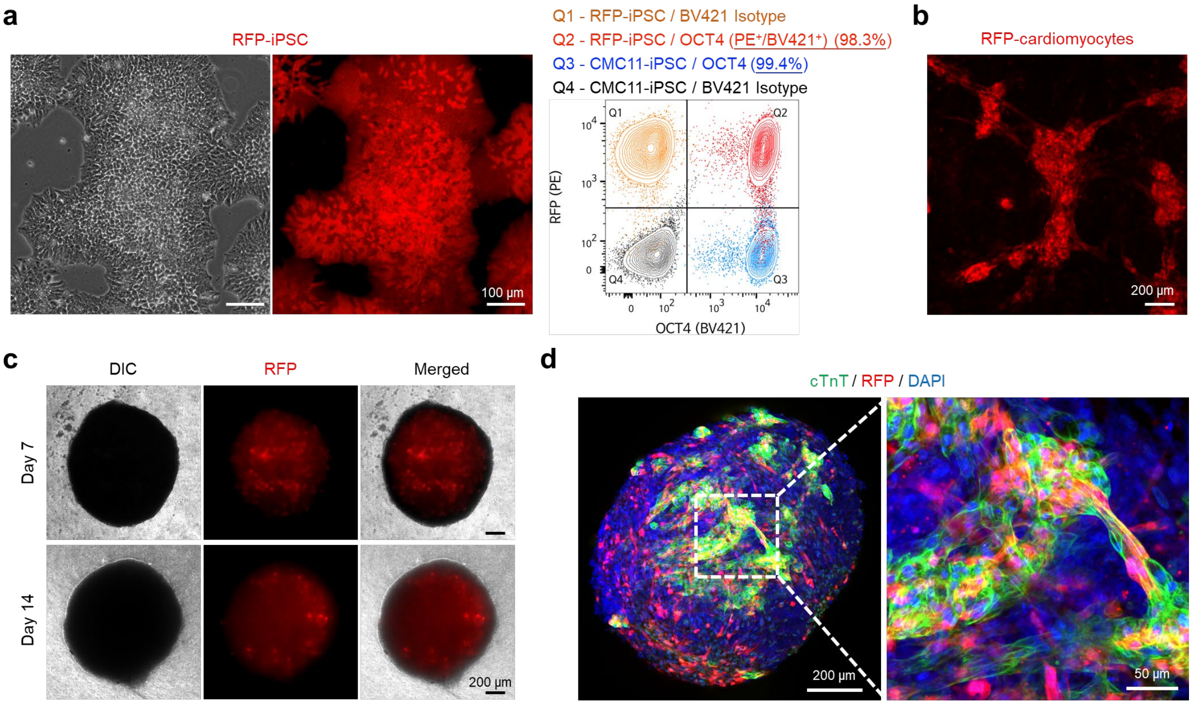

Supplementary Figure 16. Development of human cardiac tissues containing red fluorescent protein (RFP)-expressing cardiomyocytes (CMs) for transplantation. (**a**) Bright-field and fluorescent images of human induced pluripotent stem cells (hiPSCs) expressing RFP (scale bars = 100 µm). OCT4 expression in RFP-iPSCs was verified by comparing wild-type iPSCs (CMC11-iPSCs) with RFP-iPSCs using fluorescence-activated cell sorting (FACS) analysis. (**b**) Fluorescent image of CMs differentiated from RFP-iPSCs (scale bar = 200 µm). (**c**) Bright-field, fluorescent, and merged images of cardiac tissues fabricated using RFP-CMs (scale bars = 200 µm). (**d**) Immunofluorescent images of CMs (cTnT) in cardiac tissues fabricated using RFP-CMs (scale bar = 200 µm for the left image and 50 µm for the right image). Cardiac tissues were cultured for 7 days.

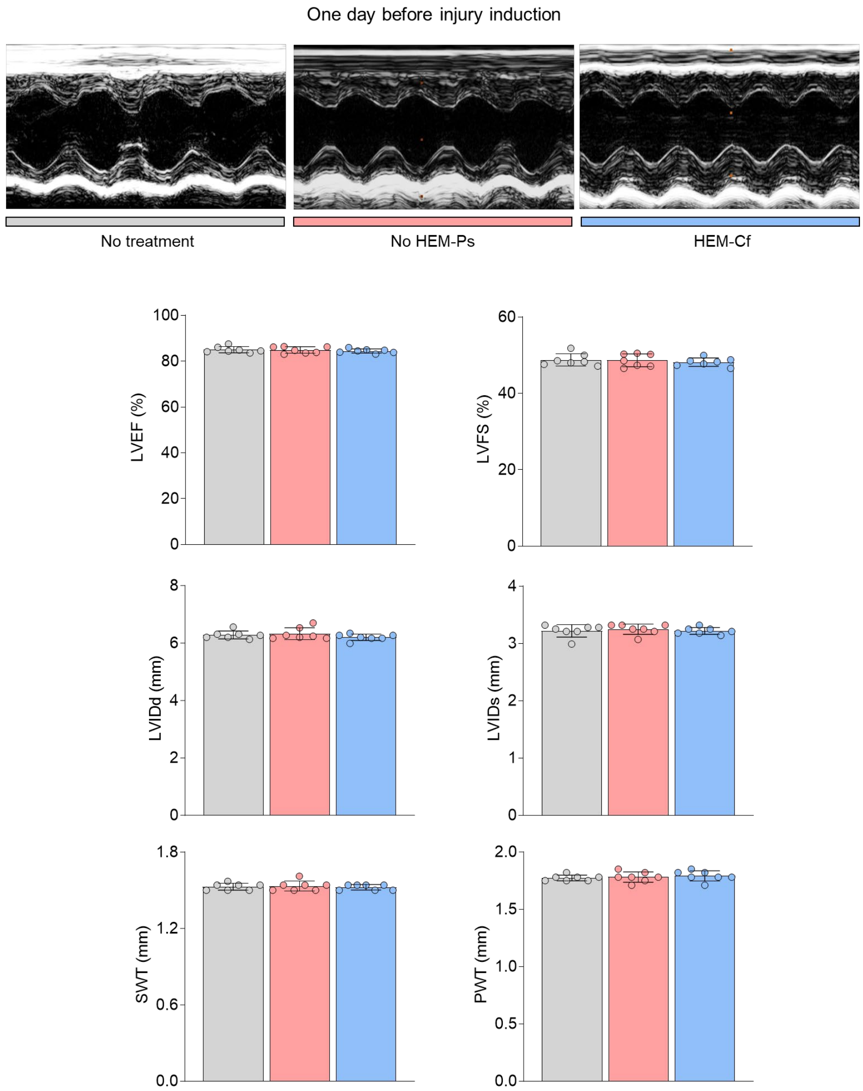

Supplementary Figure 17. Representative echocardiography images, heart functions, and left ventricular (LV) dimensions measured one day before ischemia/reperfusion injury induction. All groups (No treatment, No HEM-Ps, and HEM-Cf) showed the normal state of rats because the analysis was conducted before injury. Human cardiac tissues that were fabricated with U-bottom plate and cultured in 24-well plate under static condition (No HEM-Ps) or with heart extracellular matrix (HEM) hydrogel and cultured in microfluidic chip with flow (HEM-Cf) were transplanted to the rats allocated to each group one week after the injury. Left ventricular ejection fraction (LVEF), left ventricular fractional shortening (LVFS), left ventricular internal diameter end-diastole (LVIDd), and left ventricular internal diameter end-systole (LVIDs), septal wall thickness (SWT), and posterior wall thickness (PWT) were quantified ($N = 7$, biological replicates). Data are presented as means ± S.D.

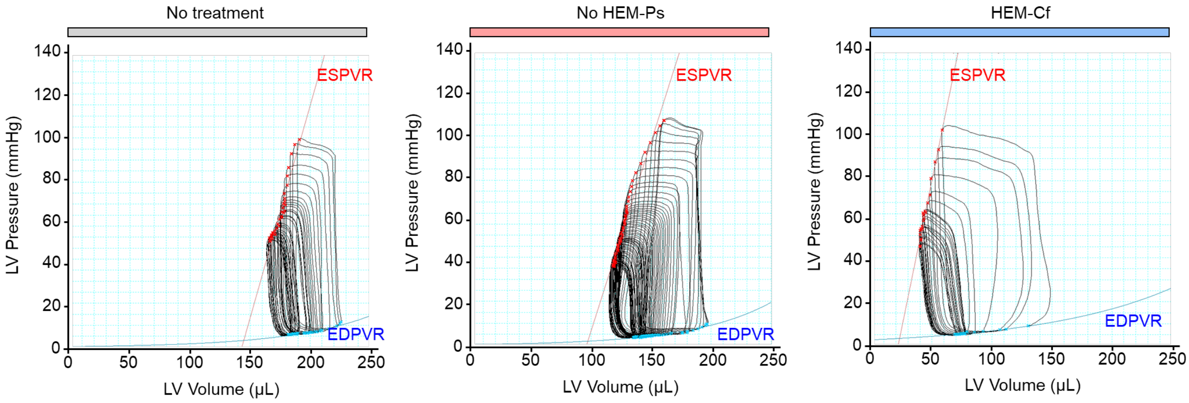

Supplementary Figure 18. Representative graphs of the slope of end-systolic pressure-volume relationship (ESPVR) and the slope of end-diastolic pressure-volume relationship (EDPVR) as measured by transient inferior vena cava (IVC) occlusion. Hemodynamic measurements were performed 4 weeks post-transplantation. For transplantation, cardiac tissues prepared with human induced pluripotent stem cell (hiPSC)-derived red fluorescent protein (RFP)$^{+}$ cardiomyocytes (CMs), cardiac fibroblasts (CFs), and human umbilical cord vein endothelial cells (HUVECs) were cultured under each condition for 9 days.

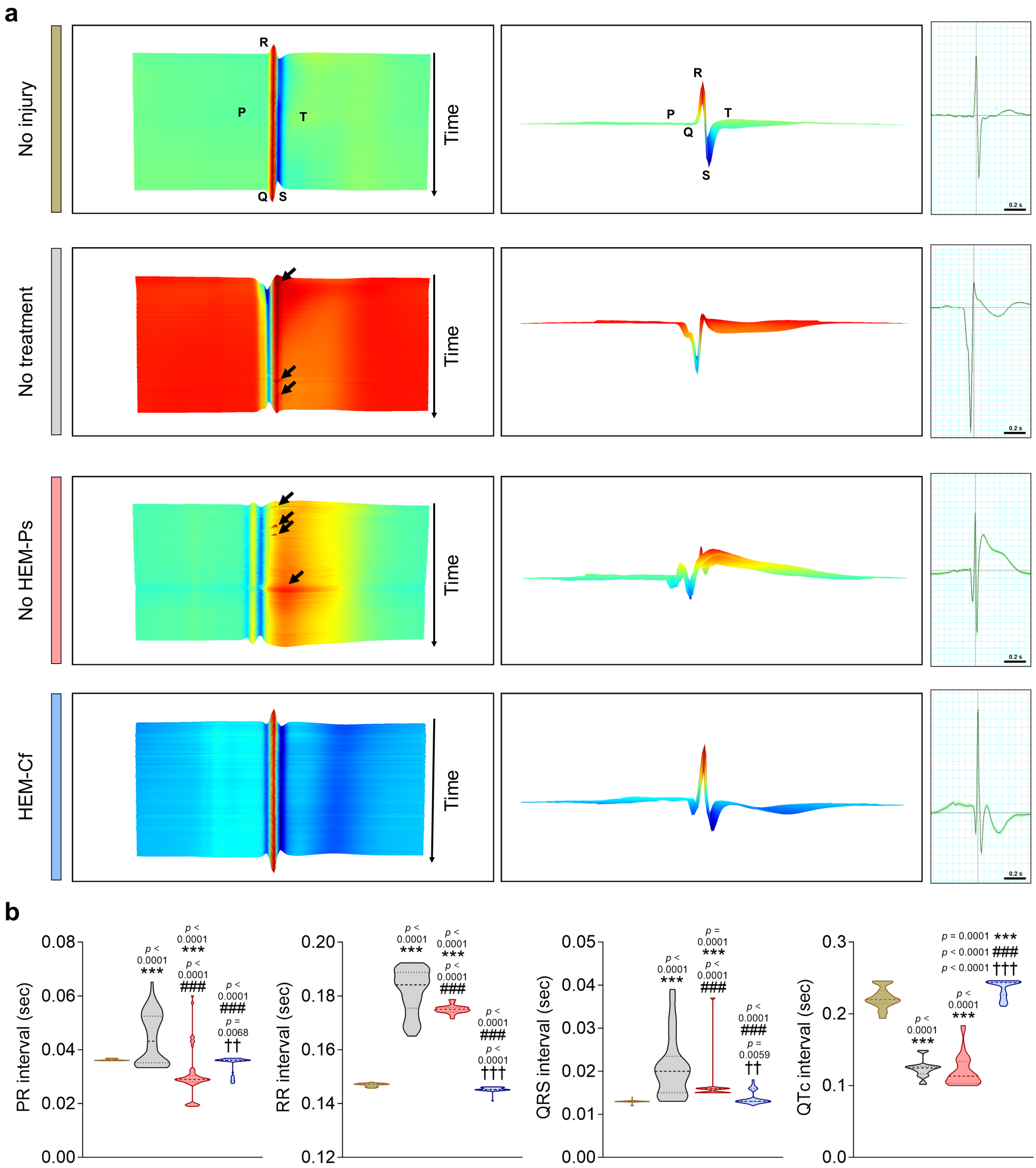

Supplementary Figure 19. Electrocardiogram (ECG) measurement using a Langendorff isolated heart perfusion system. (**a**) Representative waterfall plot mapping images of ECG recorded for 10 min at 6 weeks after transplantation. The black arrows indicate premature beats (scale bars = 0.2 sec). (**b**) The quantification of PR, RR, QRS and corrected QT (QTc) intervals recorded in each group ($N = 50$ from 5 biological replicates, $^{***}p < 0.001$ versus No injury group, $^{###}p < 0.001$ versus No treatment group, $^{††}p < 0.01$, and $^{†††}p < 0.001$ versus No HEM-Ps group). Data are presented as violin plot. Statistical significance was determined using one-way ANOVA with Tukey's multiple comparisons tests.

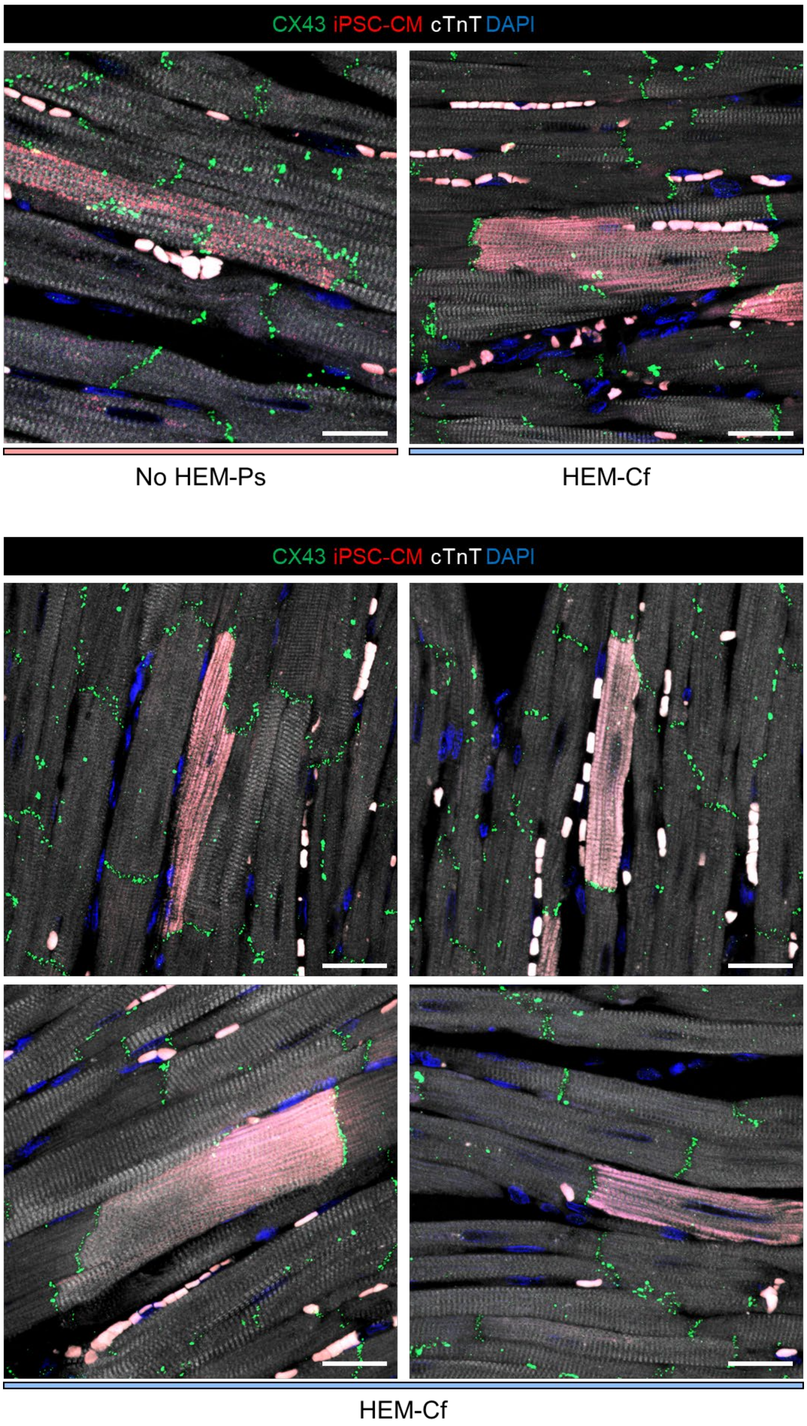

Supplementary Figure 20. High-magnification immunofluorescent images of gap junctions expressed in transplanted red fluorescent protein (RFP)$^+$ cardiomyocytes (CMs) incorporated into host CMs. Gap junctions were stained with CX43 (green) and total CMs were stained with cTnT (white) 6 weeks post-transplantation (scale bars = 100 μm).

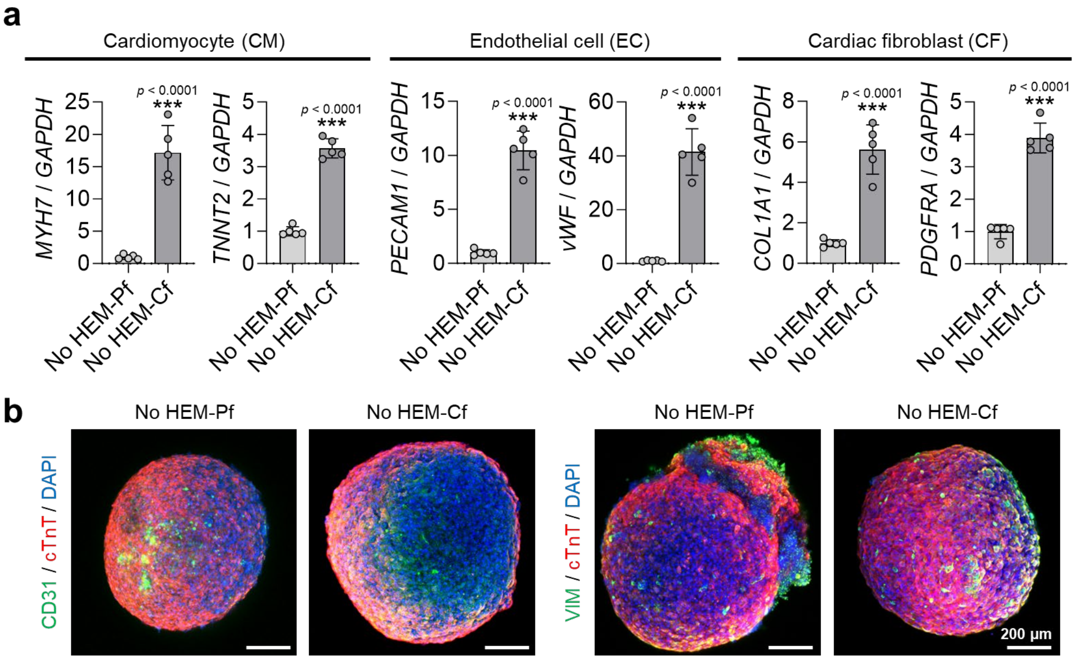

Supplementary Figure 21. The influence of dynamic flow via microfluidic chip on human cardiac tissues fabricated without heart extracellular matrix (HEM) hydrogels (No HEM). (**a**) Quantitative PCR analysis of relative mRNA expression levels of cardiomyocyte (CM; *MYH7* and *TNNT2*), endothelial cell (EC; *PECAM1* and *vWF*), and cardiac fibroblast (CF; *COL1A1* and *PDGFRA*) markers in each group of cardiac tissues ($N = 5$, biological replicates, $***p < 0.001$). (**b**) Immunofluorescent images of CM (cTnT), EC (CD31), and CF (VIM) markers in each group of cardiac tissues (scale bars = 200 μm). Cardiac tissues were cultured in 24-well plates with flow (No HEM-Pf; Plate flow) or microfluidic chips with flow (No HEM-Cf; Chip flow) for 7 days. Data are presented as means ± S.D. Statistical significance was determined using unpaired two-sided Student's *t*-tests.

REFERENCES

1. Lee, J.S. et al. Liver extracellular matrix providing dual functions of two-dimensional substrate coating and three-dimensional injectable hydrogel platform for liver tissue engineering. *Biomacromolecules* **15**, 206-218 (2014).
2. Smarr, B.L., Grant, A.D., Zucker, I., Prendergast, B.J. & Kriegsfeld, L.J. Sex differences in variability across timescales in BALB/c mice. *Biol. Sex Differ.* **8**, 7 (2017).
3. Cox, J. & Mann, M. MaxQuant enables high peptide identification rates, individualized p.p.b.-range mass accuracies and proteome-wide protein quantification. *Nat. Biotechnol.* **26**, 1367-1372 (2008).
4. UniProt, C. UniProt: the universal protein knowledgebase in 2021. *Nucleic Acids Res.* **49**, D480-D489 (2021).
5. Naba, A. et al. The extracellular matrix: Tools and insights for the "omics" era. *Matrix Biol.* **49**, 10-24 (2016).
6. Thul, P.J. et al. A subcellular map of the human proteome. *Science* **356**, eaal3321 (2017).
7. Ashburner, M. et al. Gene ontology: tool for the unification of biology. The Gene Ontology Consortium. *Nat. Genet.* **25**, 25-29 (2000).
8. Gene Ontology, C. The Gene Ontology resource: enriching a GOld mine. *Nucleic Acids Res.* **49**, D325-D334 (2021).
9. Hoang, P., Wang, J., Conklin, B.R., Healy, K.E. & Ma, Z. Generation of spatial-patterned early-developing cardiac organoids using human pluripotent stem cells. *Nat. Protoc.* **13**, 723-737 (2018).
10. Zhang, H. et al. Generation of Quiescent Cardiac Fibroblasts From Human Induced Pluripotent Stem Cells for In Vitro Modeling of Cardiac Fibrosis. *Circ. Res.* **125**, 552-566 (2019).
11. Cho, A.N. et al. Microfluidic device with brain extracellular matrix promotes structural and functional maturation of human brain organoids. *Nat. Commun.* **12**, 4730 (2021).
12. Jin, Y. et al. Vascularized Liver Organoids Generated Using Induced Hepatic Tissue and Dynamic Liver-Specific Microenvironment as a Drug Testing Platform. *Adv. Funct. Mater.* **28**, 1801954 (2018).
13. Richards, D.J. et al. Human cardiac organoids for the modelling of myocardial infarction and drug cardiotoxicity. *Nat. Biomed. Eng.* **4**, 446-462 (2020).
14. Sekine, K. et al. Oxygen consumption of human heart cells in monolayer culture. *Biochem. Biophys. Res. Commun.* **452**, 834-839 (2014).
15. Sala, L. et al. MUSCLEMOTION: A Versatile Open Software Tool to Quantify Cardiomyocyte and Cardiac Muscle Contraction In Vitro and In Vivo. *Circ. Res.* **122**, e5-e16 (2018).
16. Vandenberk, B. et al. Which QT Correction Formulae to Use for QT Monitoring? *J. Am. Heart Assoc.* **5**, e003264 (2016).
