# Supplementary material for: Versatile human cardiac tissues engineered with perfusable heart extracellular microenvironment for biomedical applications
Source: Nat Commun. 2024 Mar 22;15:2564. doi: 10.1038/s41467-024-46928-y (PMC10960018; doi:10.1038/s41467-024-46928-y)
Supplement: Supplementary file 3 — Description of additional supplementary files [file 41467_2024_46928_MOESM3_ESM.pdf]

Description of Additional supplementary files

Supplementary Data 1 : The lists of total proteins contained in HEM samples derived from porcine A#1, A#2, A#3, B, and C that were detected by proteomic analysis.

Supplementary Movie 1 : The movie showing synchronized contractions of spontaneously beating human cardiac tissue (HEM-Cf group) on day 14 after fabrication.

Supplementary Movie 2 : The movie showing synchronized Ca2+ transients of spontaneously beating human cardiac tissue (HEM-Cf group) on day 14 after fabrication.
