# Supplementary material for: Versatile human cardiac tissues engineered with perfusable heart extracellular microenvironment for biomedical applications
Source: Nat Commun. 2024 Mar 22;15:2564. doi: 10.1038/s41467-024-46928-y (PMC10960018; doi:10.1038/s41467-024-46928-y)
Supplement: Supplementary file 7 — Reporting Summary [file 41467_2024_46928_MOESM7_ESM.pdf]

nature portfolio

Corresponding author(s): Seung-Woo Cho

Last updated by author(s): Feb 15, 2024

Reporting Summary

Nature Portfolio wishes to improve the reproducibility of the work that we publish. This form provides structure for consistency and transparency in reporting. For further information on Nature Portfolio policies, see our Editorial Policies and the Editorial Policy Checklist.

Statistics

For all statistical analyses, confirm that the following items are present in the figure legend, table legend, main text, or Methods section.

n/a	Confirmed	
☐	☒	The exact sample size (*n*) for each experimental group/condition, given as a discrete number and unit of measurement
☐	☒	A statement on whether measurements were taken from distinct samples or whether the same sample was measured repeatedly
☐	☒	The statistical test(s) used AND whether they are one- or two-sided<br>*Only common tests should be described solely by name; describe more complex techniques in the Methods section.*
☒	☐	A description of all covariates tested
☐	☒	A description of any assumptions or corrections, such as tests of normality and adjustment for multiple comparisons
☐	☒	A full description of the statistical parameters including central tendency (e.g. means) or other basic estimates (e.g. regression coefficient) AND variation (e.g. standard deviation) or associated estimates of uncertainty (e.g. confidence intervals)
☐	☒	For null hypothesis testing, the test statistic (e.g. *F*, *t*, *r*) with confidence intervals, effect sizes, degrees of freedom and *P* value noted<br>*Give P values as exact values whenever suitable.*
☒	☐	For Bayesian analysis, information on the choice of priors and Markov chain Monte Carlo settings
☐	☒	For hierarchical and complex designs, identification of the appropriate level for tests and full reporting of outcomes
☐	☒	Estimates of effect sizes (e.g. Cohen's *d*, Pearson's *r*), indicating how they were calculated

Our web collection on statistics for biologists contains articles on many of the points above.

Software and code

Policy information about availability of computer code

Data collection	Microscopy: Ocular 2.0<br>Confocal microscopy: Zeiss Zen 3.0<br>Microplate reader: i-control.2.0<br>qPCR: StepOne™ Software 2.3
Data analysis	Quantification data and statistics: Microsoft Excel (Office 365, 16.0), GraphPad Prism 10.1.2<br>Imaging: Microsoft PowerPoint (Office 365, 16.0), Zeiss Zen 3.0, ImageJ 1.51j8, OlyVIA 2.9<br>Proteomics: MaxQuant (2.0.3.0), Perseus software 1.6.10.43, g:Profiler (version e104_eg51_p15_3922dba)<br>RNA sequencing: Tophat (v2.0.13), Cuffdiff (v2.2.1), Cluster 3.0, Java TreeView 1.2.0, Rstudio (2022.02.3), ggfortify and ggplot2 R package (version 3.3.5)<br>Contractility: MUSCLEMOTION plugin for ImageJ, SoftEdgeTM Acquisition software<br>Computational simulation: COMSOL Multiphysics software<br>MEA: AxIS software (version 2.3.2.4), Synapse Suite (version 94)<br>FACS: FlowJo software (10.8.1)<br>ECG: AD instruments Labchart

For manuscripts utilizing custom algorithms or software that are central to the research but not yet described in published literature, software must be made available to editors and reviewers. We strongly encourage code deposition in a community repository (e.g. GitHub). See the Nature Portfolio guidelines for submitting code & software for further information.

Data

Policy information about availability of data

All manuscripts must include a data availability statement. This statement should provide the following information, where applicable:

- Accession codes, unique identifiers, or web links for publicly available datasets
- A description of any restrictions on data availability
- For clinical datasets or third party data, please ensure that the statement adheres to our policy

All datasets supporting this study are available within this article and its supplementary information file. Proteomics data are available at the ProteomeXchange Consortium via the PRIDE partner repository with the dataset identifier "PXD042077" [proteomecentral.proteomexchange.org/cgi/GetDataset?ID=PXD042077]. Proteomics data from LC-MS/MS were processed using the UniProt database [uniprot.org]. Matrisome proteins were identified and categorized using the Matrisome Project database [sites.google.com/uic.edu/matrisome/]. Heart-enriched proteins were identified using the Human Protein Atlas [proteinatlas.org]. RNA-sequencing data have been deposited to the Gene Expression Omnibus (GEO) public repository under accession codes "GSE231493" [ncbi.nlm.nih.gov/geo/query/acc.cgi?acc=GSE231493]. All raw data from quantitative analyses in our study are provided as a single Excel file 'Source Data'. All microscopic images and other data generated for this study are available from the corresponding author on reasonable request, because they are too large and complicated to be deposited. Source data are provided with this paper.

Research involving human participants, their data, or biological material

Policy information about studies with human participants or human data. See also policy information about sex, gender (identity/presentation), and sexual orientation and race, ethnicity and racism.

Reporting on sex and gender	Not applicable
Reporting on race, ethnicity, or other socially relevant groupings	Not applicable
Population characteristics	Not applicable
Recruitment	Not applicable
Ethics oversight	Not applicable

Note that full information on the approval of the study protocol must also be provided in the manuscript.

Field-specific reporting

Please select the one below that is the best fit for your research. If you are not sure, read the appropriate sections before making your selection.

☒ Life sciences ☐ Behavioural & social sciences ☐ Ecological, evolutionary & environmental sciences

For a reference copy of the document with all sections, see nature.com/documents/nr-reporting-summary-flat.pdf

Life sciences study design

All studies must disclose on these points even when the disclosure is negative.

Sample size	No statistical method was used to predetermine the sample size. Throughout the study, at least three samples were included for one experiment to ensure sufficient reproducibility of the results. Biological replicates (N) and the numbers of independent experiment are indicated in the figure legends. At least 3 biological samples were included for one experiment and 1 to 3 independent experiments were performed to ensure sufficient reproducibility of the results.
Data exclusions	No data were excluded from the analysis.
Replication	The number of experiments performed in the study is noted in each figure legend. Similar results were observed in all repeated trials for each experiment.
Randomization	Cardiac tissues were generated under the same conditions with the same cell numbers, and they were randomly assigned for analysis when they reached the specified time points in each experiment. For testing the in vivo biocompatibility of HEM hydrogel, the mice were randomly allocated to each experimental group. For cardiac tissue transplantation, the rats were randomly allocated to each group after ischemic injury. Likewise, samples of all other experiments were randomly allocated to each group.
Blinding	The collection of all proteomics and RNA-sequencing data was performed in blind. In the echocardiography for evaluation of infarcted heart function after cardiac tissue transplantation, the operator was blinded to the group allocation during the experiment. For other analyses, the identities of the samples were not blinded to investigators. However, the investigators collected and analyzed the data from all experimental and control groups under the same conditions and at the same time, and performed the analysis with the same software setting to avoid any potential bias due to lack of blinding.

Reporting for specific materials, systems and methods

We require information from authors about some types of materials, experimental systems and methods used in many studies. Here, indicate whether each material, system or method listed is relevant to your study. If you are not sure if a list item applies to your research, read the appropriate section before selecting a response.

Materials & experimental systems

n/a	Involved in the study
☐	☒ Antibodies
☐	☒ Eukaryotic cell lines
☒	☐ Palaeontology and archaeology
☐	☒ Animals and other organisms
☒	☐ Clinical data
☒	☐ Dual use research of concern
☒	☐ Plants

Methods

n/a	Involved in the study
☒	☐ ChIP-seq
☐	☒ Flow cytometry
☒	☐ MRI-based neuroimaging

Antibodies

Antibodies used

The following primary antibodies were used:
mouse monoclonal (G-4) anti-GATA-4 (#sc-25310, 1:200, Santa Cruz Biotechnology)
mouse monoclonal (EA-53) anti-α-actinin (#A7811, 1:400, Sigma-Aldrich)
rabbit polyclonal anti-cardiac troponin T (#ab45932, 1:400, Abcam)
mouse monoclonal (13-11) anti-cardiac troponin T (#MA5-12960, 1:100, Thermo Fisher Scientific)
mouse monoclonal (1C11) anti-cardiac troponin T (#ab8295, 1:200, Abcam)
rabbit polyclonal anti-cardiac troponin I (cTnI, #ab47003, 1:200, Abcam)
rabbit polyclonal anti-connexin-43 (CX43, #C6219, 1:200, Sigma-Aldrich)
rabbit polyclonal anti-connexin-43 (#ab11370, 1:300, Abcam)
mouse monoclonal (9G11) anti-CD31 (#BBA7, 10 μg/ml, R&D Systems)
rabbit polyclonal anti-CD31 (#ab28364, 1:200, Abcam)
mouse monoclonal (3B11E4) anti-DDR2 (#sc-81707, 1:200, Santa Cruz Biotechnology)
mouse monoclonal (LN-6) anti-vimentin (VIM, #MAB1681, 1:100, Millipore Corporation)
rabbit polyclonal anti-collagen type 1 (#234167, 1:200, Millipore Corporation)
mouse monoclonal (B4) anti-α-smooth muscle actin (α-SMA, #sc-53142, 1:200, Santa Cruz Biotechnology)
rat monoclonal (M1/70) anti-CD11b (#ab8878, 1:200, Abcam)
mouse polyclonal anti-CD45 (#AF114, 200 μg/ml, R&D Systems)
mouse monoclonal (C-11) anti-inducible nitric oxide synthase (iNOS, #sc7271, 1:200, Santa Cruz Biotechnology)
rabbit polyclonal anti-CD206 (#ab64693, 1:200, Abcam)
mouse monoclonal (8-E5) anti-CD80 (#MA5-42562, 1:100, Thermo Fisher Scientific)
rabbit monoclonal (EPR19518) anti-CD163 (#ab182422, 1:200, Abcam)
rabbit polyclonal anti-cleaved caspase-3 (#9661S, 1:400, Cell Signaling Technology)
BV421 mouse monoclonal (40/Oct-3) anti-Oct3/4 (565644, BD Biosciences)
BV421 mouse monoclonal (X40) IgG1, k Isotype control (562438, BD Biosciences)

The following secondary antibodies were used:
anti-mouse Alexa Fluor 488 (#A11001, 1:200, Thermo Fisher Scientific)
anti-rabbit Alexa Fluor 488 (#A11008, 1:200, Thermo Fisher Scientific)
anti-mouse Alexa Fluor 488 (#A21202, 1:500, Thermo Fisher Scientific)
anti-rabbit Alexa Fluor 488 (#A21206, 1:500, Thermo Fisher Scientific)
anti-rat Alexa Fluor 488 (#A11006, 1:200, Thermo Fisher Scientific)
anti-rabbit Alexa Fluor 555 (#4413S, 1:500, Cell Signaling Technology)
anti-mouse Alexa Fluor 594 (#A11005, 1:200, Thermo Fisher Scientific)
anti-rabbit Alexa Fluor 594 (#A11012, 1:200, Thermo Fisher Scientific)
anti-rabbit Alexa Fluor 647 (#A31573, 1:500, Thermo Fisher Scientific)

Validation

All antibodies listed above are commercially available and have been verified by many references provided on the website of the companies that sell antibodies (links below).

mouse monoclonal (G-4) anti-GATA-4 (#sc-25310, 1:200, Santa Cruz Biotechnology)
Validation Refs. from the manufacturer's datasheet: https://datasheets.scbt.com/sc-25310.pdf

mouse monoclonal (EA-53) anti-α-actinin (#A7811, 1:400, Sigma-Aldrich)
Validation Refs. from the manufacturer's datasheet: https://www.sigmaaldrich.com/KR/en/product/sigma/a7811

rabbit polyclonal anti-cardiac troponin T (#ab45932, 1:400, Abcam)
Validation Refs. from the manufacturer's datasheet: https://www.abcam.com/products/primary-antibodies/cardiac-troponin-t-antibody-ab45932.html

mouse monoclonal (13-11) anti-cardiac troponin T (#MA5-12960, 1:100, Thermo Fisher Scientific)

Validation Refs. from the manufacturer's datasheet: https://www.thermofisher.com/antibody/product/Cardiac-Troponin-T-Antibody-clone-13-11-Monoclonal/MA5-12960

mouse monoclonal (1C11) anti-cardiac troponin T (#ab8295, 1:200, Abcam)
Validation Refs. from the manufacturer's datasheet: https://www.abcam.com/products/primary-antibodies/cardiac-troponin-t-antibody-1c11-ab8295.html

rabbit polyclonal anti-cardiac troponin I (cTnI, #ab47003, 1:200, Abcam)
Validation Refs. from the manufacturer's datasheet: https://www.abcam.com/products/primary-antibodies/cardiac-troponin-i-antibody-ab47003.html

rabbit polyclonal anti-connexin-43 (CX43, #C6219, 1:200, Sigma-Aldrich)
Validation Refs. from the manufacturer's datasheet: https://www.sigmaaldrich.com/KR/en/product/sigma/c6219

rabbit polyclonal anti-connexin-43 (#ab11370, 1:300, Abcam)
Validation Refs. from the manufacturer's datasheet: https://www.abcam.com/products/primary-antibodies/connexin-43--gja1-antibody-intercellular-junction-marker-ab11370.html

mouse monoclonal (9G11) anti-CD31 (#BBA7, 10 µg/ml, R&D Systems)
Validation Refs. from the manufacturer's datasheet: https://www.rndsystems.com/products/human-cd31-pecam-1-antibody-9g11_bba7

rabbit polyclonal anti-CD31 (#ab28364, 1:200, Abcam)
Validation Refs. from the manufacturer's datasheet: https://www.abcam.com/products/primary-antibodies/cd31-antibody-ab28364.html

mouse monoclonal (3B11E4) anti-DDR2 (#sc-81707, 1:200, Santa Cruz Biotechnology)
Validation Refs. from the manufacturer's datasheet: https://datasheets.scbt.com/sc-81707.pdf

mouse monoclonal (LN-6) anti-vimentin (VIM, #MAB1681, 1:100, Millipore Corporation)
Validation Refs. from the manufacturer's datasheet: https://www.merckmillipore.com/product/Anti-Vimentin-Antibody-clone-LN-6,MM_NF-MAB1681

rabbit polyclonal anti-collagen type 1 (#234167, 1:200, Millipore Corporation)
Validation Refs. from the manufacturer's datasheet: https://www.merckmillipore.com/product/Anti-Collagen-Type-I-Rabbit-pAb,EMD_BIO-234167

mouse monoclonal (B4) anti-α-smooth muscle actin (α-SMA, #sc-53142, 1:200, Santa Cruz Biotechnology)
Validation Refs. from the manufacturer's datasheet: https://datasheets.scbt.com/sc-53142.pdf

rat monoclonal (M1/70) anti-CD11b (#ab8878, 1:200, Abcam)
Validation Refs. from the manufacturer's datasheet: https://www.abcam.com/products/primary-antibodies/cd11b-antibody-m170-ab8878.html

mouse polyclonal anti-CD45 (#AF114, 200 µg/ml, R&D Systems)
Validation Refs. from the manufacturer's datasheet: https://www.rndsystems.com/products/mouse-cd45-antibody_af114

mouse monoclonal (C-11) anti-inducible nitric oxide synthase (iNOS, #sc7271, 1:200, Santa Cruz Biotechnology)
Validation Refs. from the manufacturer's datasheet: https://datasheets.scbt.com/sc-7271.pdf

rabbit polyclonal anti-CD206 (#ab64693, 1:200, Abcam)
Validation Refs. from the manufacturer's datasheet: https://www.abcam.com/products/primary-antibodies/mannose-receptor-antibody-ab64693.html

mouse monoclonal (8-E5) anti-CD80 (#MA5-42562, 1:100, Thermo Fisher Scientific)
Validation Refs. from the manufacturer's datasheet: https://www.thermofisher.com/antibody/product/CD80-B7-1-Antibody-clone-8-E5-Monoclonal/MA5-42562

rabbit monoclonal (EPR19518) anti-CD163 (#ab182422, 1:200, Abcam)
Validation Refs. from the manufacturer's datasheet: https://www.abcam.com/products/primary-antibodies/cd163-antibody-epr19518-ab182422.html

rabbit polyclonal anti-cleaved caspase-3 (#9661S, 1:400, Cell Signaling Technology)
Validation Refs. from the manufacturer's datasheet: https://www.cellsignal.com/products/primary-antibodies/cleaved-caspase-3-asp175-antibody/9661?&print=true

BV421 mouse monoclonal (40/Oct-3) anti-Oct3/4 (565644, BD Biosciences)
Validation Refs. from the manufacturer's datasheet: https://www.bdbiosciences.com/en-us/products/reagents/flow-cytometry-reagents/research-reagents/single-color-antibodies-ruo/bv421-mouse-anti-oct3-4.565644

BV421 mouse monoclonal (X40) IgG1, k Isotype control (562438, BD Biosciences)
Validation Refs. from the manufacturer's datasheet: https://www.bdbiosciences.com/en-us/products/reagents/flow-cytometry-reagents/research-reagents/flow-cytometry-controls-and-lysates/bv421-mouse-igg1-k-isotype-control.562438

Eukaryotic cell lines

Policy information about cell lines and Sex and Gender in Research

Cell line source(s)	Human induced pluripotent stem cells (hiPSCs, line KYOU-DXR0109B (ACS-1023), female) were obtained from American Type Culture Collection (ATCC), and Long QT Syndrome 2 (LQT2) patient-derived hiPSCs (line GM25305, female) were obtained from Coriell Institute. Red fluorescence protein (RFP)-expressing hiPSCs (CMC-iPSC-011, male) were obtained from the Catholic iPSC Research Center in the Catholic University of Korea. Human umbilical vein endothelial cells (HUVECs) were purchased from Lonza. The studies involving these cell lines were approved by the Institutional Review Board (IRB) of Yonsei University (permit number: 7001988-202004-BR-844-01E).
Authentication	hiPSCs were authenticated with immunostaining of pluripotency markers (OCT4, TRA-1-60, SOX2) and alkaline phosphatase staining. HUVECs were authenticated by Lonza before delivery and not authenticated subsequently.
Mycoplasma contamination	hiPSCs were routinely tested and negative for mycoplasma contamination. HUVECs negative for mycoplasma contamination were purchased and not authenticated subsequently.
Commonly misidentified lines (See ICLAC register)	No misidentified cell lines were used in this study.

Animals and other research organisms

Policy information about studies involving animals; ARRIVE guidelines recommended for reporting animal research, and Sex and Gender in Research

Laboratory animals	BALB/c nude mice (CAnN.Cg-Foxn1 nu/CrlOri, male, 4-weeks-old and 20 g; Orient Bio Inc., Seongnam, Korea) were used for HEM biocompatibility test. Fischer 344 rats (F344/NHsd, male, 8-weeks-old and 160 to 180 g; KOATECH, Pyeongtaek, Korea) were used for myocardial infarction model. All mice and rats were maintained in the housing condition with a temperature of 21±2°C, a humidity of 50±10%, ventilation of 10–15/h, the light of 150–300 Lux, and noise of less than 60 dB.
Wild animals	Porcine tissues for decellularization were freshly obtained from a local market. No wild animals were used in the study.
Reporting on sex	No sex-based analysis was performed in this study. For decellularization, female pigs were utilized because they are overwhelmingly more common in the marketplace. For HEM biocompatibility test, male mice were employed to minimize variation of results according to biological sex differences. For MI model, male rats were employed to minimize variation in the outcomes of ischemia/reperfusion injury, considering biological sex.
Field-collected samples	No field-collected samples were used in this study.
Ethics oversight	Animal study for HEM biocompatibility test was approved by the Institutional Animal Care and Use Committee (IACUC) of the Yonsei Laboratory Animal Research Center (YLARC) (permit number: IACUC-A-202111-1373-04). Animal study for cardiac tissue transplantation was approved by the IACUC of the Catholic University of Korea (approval number: CUMC-2021-0135-05). These animal procedures were conformed to the NIH guidelines or the guidelines issued by Directive 2010/63/EU of the European Parliament for the protection of animals used in scientific research.

Note that full information on the approval of the study protocol must also be provided in the manuscript.

Plants

Seed stocks	Not applicable
Novel plant genotypes	Not applicable
Authentication	Not applicable

Flow Cytometry

Plots

Confirm that:

☒ The axis labels state the marker and fluorochrome used (e.g. CD4-FITC).

☒ The axis scales are clearly visible. Include numbers along axes only for bottom left plot of group (a 'group' is an analysis of identical markers).

☒ All plots are contour plots with outliers or pseudocolor plots.

☒ A numerical value for number of cells or percentage (with statistics) is provided.

Methodology

Sample preparation	hiPSCs were transfected with a tdTomato expression plasmid, followed by antibiotic selection. The cells were harvested with Accutase solution and subjected to Fix/Perm using BD Cytofix™ Fixation Buffer and Phosflow™ Perm Buffer III. Staining was conducted using BV421 mouse anti-Oct3/4 (565644, BD Biosciences) or BV421 mouse IgG1, k Isotype control (562438, BD Biosciences).
Instrument	FACSAria™ Fusion (BD Biosciences) and FACSCanto II (BD Biosciences)
Software	Data analysis was performed using FlowJo 10.8.1
Cell population abundance	The abundance of the post-sort fractions was higher than 98%.
Gating strategy	First gating was performed through FSC-A/SSC-A, followed by selecting a single population through FSC-A/FSC-H. The expression level of OCT4 was then calculated based on the BV421 Isotype control. For confirmation of RFP expression, a parental cell line without tdTomato expression was used as a control.

☒ Tick this box to confirm that a figure exemplifying the gating strategy is provided in the Supplementary Information.
